# *Tert* Deletion Impairs Circadian Regulation of Blood Pressure in Male Spontaneously Hypertensive Rats

**DOI:** 10.1161/HYPERTENSIONAHA.125.25510

**Published:** 2025-12-10

**Authors:** Kateryna Semenovykh, Michal Pravenec, Ivana Vaněčková, Pavel Houdek, Martin Sládek, Miroslava Šimáková, Petr Mlejnek, Saba Selvi, Jan Šilhavý, František Liška, Dmytro Semenovykh, Silvie Hojná, Alena Sumová

**Affiliations:** Laboratory of Biological Rhythms (K.S., P.H., M.S., S.S., D.S., A.S.), Institute of Physiology, Czech Academy of Sciences, Prague.; Laboratory of Genetics of Model Diseases (M.P., M.Š., P.M., J.Š.., F.L.), Institute of Physiology, Czech Academy of Sciences, Prague.; Laboratory of Experimental Hypertension (I.V., S.H.), Institute of Physiology, Czech Academy of Sciences, Prague.; First Faculty of Medicine, Institute of Biology and Medical Genetics, Charles University and General University Hospital, Prague, Czech Republic (M.P., F.L.).

**Keywords:** blood pressure, circadian clocks, heart rate, oxidative stress, rats

## Abstract

**BACKGROUND:**

Deletion of the *Tert* gene leads to telomere shortening, which is associated with aging and age-related cardiovascular disease, but its effects on circadian regulation of blood pressure have not yet been investigated. To fill this gap, we developed a rat model with genetic deletion of the *Tert* gene on a spontaneously hypertensive rat-*Tert*^−/−^ background, in which telomeres were shortened in the F3 generation.

**METHODS:**

We analyzed the effects of *Tert* deletion on locomotor activity, oxidative stress, telemetrically measured parameters of the cardiovascular system, and the expression of clock genes in various tissues.

**RESULTS:**

Male spontaneously hypertensive rat-*Tert*^−/−^ showed a reduced physical fitness, which was reflected in a more fragmented nocturnal activity and a poorer correlation between spontaneous activity and cardiovascular parameters. Day/night blood pressure amplitude was reduced, and the circadian rhythm of systolic blood pressure was completely abolished in constant darkness. In the rostral ventromedial medulla of the brainstem, the number of TH (tyrosine hydroxylase)-immunopositive cells was reduced, indicating a decrease in sympathetic tone. In heart tissue, the level of protein oxidation was increased, and similar to other tissues, the day/night expression of clock genes was significantly changed, suggesting an impairment of the synchrony of their clocks.

**CONCLUSIONS:**

Our results suggest that deletion of *Tert* impairs circadian regulation of blood pressure via a decreased rhythm of sympathetic activity innervating the heart and other tissues, leading to impaired circadian regulation of local peripheral clocks. These findings provide a possible link between age-related telomere shortening and impaired rhythmicity of cardiovascular function.

**NOVELTY AND RELEVANCE**

**What Is New?**

We have created a new genetic rat model with deletion of the *Tert* gene in which the F3 generation showed shortened telomeres in various tissues. The rats exhibited signs of accelerated aging at the behavioral level although the circadian regulation of locomotor activity was not impaired. In the rostral ventromedial medulla of the brainstem, the number of TH (tyrosine hydroxylase)-immunopositive cells was reduced, indicating a decrease in sympathetic tone. Importantly, the day/night rhythmicity of clock gene expression in the heart and other peripheral tissues was modulated in constant darkness, which was reflected in the complete abolition of the daily rhythm of heart rate and blood pressure. This is the first study to show the effects of deletion of *Tert* on peripheral circadian clocks and regulation of cardiovascular function at a systemic level.

**What Is Relevant?**

Telomere shortening is associated with the aging process. The gene was deleted in spontaneously hypertensive rats, which spontaneously develop hypertension and are closely associated with higher susceptibility to circadian dysregulation. Comparison of spontaneously hypertensive rat-*Tert*^−/−^ with control spontaneously hypertensive rat showed that the gene is involved in the circadian regulation of cardiovascular functions in this species, as its deletion abolished the rhythm of heart rate and blood pressure.

**Clinical/Pathophysiological Implications?**

The mechanisms of age-related pathophysiology are still largely unknown. Given the increasing longevity and age-related diseases in the human population, advances in this area of research could help to extend the interval of healthy aging.

The complexity of the interaction between the circadian and cardiovascular systems has been well documented.^1^ Both systems are progressively affected by aging, resulting in an impairment of the circadian rhythm of blood pressure (BP) due to the lack of its decrease at night (nondipper), which is a known factor increasing the risk of cardiovascular disease.^2^ The mechanism of the age-dependent effects is not fully understood. One of the major hallmarks of aging is telomere shortening due to loss of activity of the enzyme TERT (telomerase reverse transcriptase),^3^ which is involved in the modulation of tissue repair and regeneration processes.^4^ Loss of TERT activity negatively affects cellular regulation in various tissues, including cells of the cardiovascular system with a high turnover rate, which have higher telomerase activity, such as cardiomyocytes, regenerating endothelial cells, and cardiac stem cells.^5,6^ The age-dependent decrease in *Tert* expression and TERT activity in cardiac stem cells has been documented^7^ and is associated with the development of age-related cardiovascular disease.^8,9^ The mechanism likely involves oxidative stress, which increases with age and accompanies the cardiac disease.^10,11^ Indeed, telomere shortening is considered to be associated with oxidative stress,^12,13^ and there is an extensive literature on the involvement of myocardial oxidative stress in pathological cardiac remodeling.^14,15^

Circadian regulation of BP results from the function of the circadian system, which consists of the central clock in the suprachiasmatic nuclei (SCN) of the hypothalamus that coordinates cellular clocks located throughout the body.^16^ The SCN interacts with a regulatory center of the autonomic nervous system located in the paraventricular nuclei of the hypothalamus.^17^ The PVN projects downstream to brainstem regions, including the rostral ventrolateral medulla (RVLM), a key area for regulation of arterial BP,^18,19^ and, via sympathetic autonomic nerves, controls clocks in peripheral tissues involved in cardiovascular functions.^20^ In addition to control by the autonomous nervous system, BP is regulated by various other mechanisms, including hormonal and renal regulation (for review, see the study by Guyton et al^21^).

The clocks operate via an autonomous transcriptional-translational feedback loop mechanism involving a set of clock genes (namely, *Per1,2*, *Cry1,2*, *Bmal1*, *Nr1d1,2*, *Rora,b*, and *Clock*), whose protein products accumulate at a specific time of day, bind their promoters, and regulate (activate or inhibit) their transcription. The functional operation of the clock depends on the proper mutual phasing of the individual clock gene expression rhythms.^22^ Importantly, the rhythmically oscillating clock proteins can bind promoters of many other genes, resulting in their rhythmic expression patterns. In healthy aging, the ability to generate circadian signals by the molecular core clock mechanism does not seem to be severely impaired at the level of the central and peripheral clocks.^23,24^ Nevertheless, the aged clock becomes less resilient to disruption of external cycles^23,25,26^ and consequently loses its ability to drive high-amplitude output rhythms in many physiological and cellular processes.^24^ The mechanism underlying the age-dependent effects has not yet been identified.

The relationship between TERT and the circadian clock has been investigated.^27^ While circadian regulation of *Tert* mRNA expression has been demonstrated via the binding of the clock proteins, CLOCK (circadian locomotor output cycles kaput) and BMAL1 (brain and muscle ARNT-like 1) to the *Tert* promoter, resulting in a 24-hour rhythm of TERT activity in mammals,^28^ there are only few studies on the feedback effect of TERT on the transcription-translation feedback loop. Two earlier studies reported reconstitution of the amplitudes in clock gene expression in vitro in aged vascular human smooth muscle cells^29^ and fibroblasts^30^ upon genetic rescue of TERT. However, whether the deletion of *Tert* affects peripheral circadian clocks and the regulation of cardiovascular function at the systemic level has not been shown. To fill this gap, we created a *Tert* knockout model on the spontaneously hypertensive rat (SHR) background and analyzed the impact of the *Tert* gene deletion and telomere shortening on the circadian regulation of cardiovascular function.

## Methods

### Data Availability

Detailed materials and methods can be found in the Supplemental Material. The data that support the findings of this study are available from the corresponding author upon reasonable request.

### Statistical Analyses

Data were analyzed in GraphPad Prism 8 using the *t* test or 2-way ANOVA with a Holm-Šídák post hoc multiple comparison test. *P*<0.05 was considered statistically significant. Due to the presence of the missing values caused by sensor issues in telemetry, a mixed effects model was fitted instead of the 2-way ANOVA. To evaluate the rhythmicity in telemetry data, data sets with Benjamini-Hochberg adjusted *P* value (pBH) <0.001 were considered as rhythmic, data sets with pBH> 0.001 were considered as nonrhythmic.

## Results

### Generation of SHR-*Tert*^−/−^ Rats

Using the Zinc Finger Nuclease (ZFN) technique, we generated 2 founders with mutations in *Tert* exon 1 (Figure S1A). One founder (59) had a 3-bp duplication (NM_053423.2:c.87_89dup) resulting in an in-frame insertion of an arginine moiety-p.(Arg30dup). We did not breed this line as the mutation is not predicted to abolish the protein function. The second founder (63) had an 11-bp deletion (NM_053423.2:c.83_93del), which predicts a frameshift-p.(Val28fs), with subsequent protein truncation or nonsense-mediated RNA decay and, therefore, complete loss of function (Figure S1B). Breeding this founder with SHR and intercrossing the heterozygotes generated all genotypes (Figure S1C) in the expected Mendelian ratios (not shown). The *Tert* rat sequence is provided in Data File S1. We additionally confirmed the presence of the same *Tert* deletion in the mRNA product in SHR-*Tert*^−/−^ animals using next-generation sequencing^31^ (Figure S1D). In SHR-*Tert*^−/−^ of the third generation (F3), telomere size was shortened in the spleen, liver, kidney, heart, and intestine compared with wild-type SHR (Figure S1E; Table S1).

### *Tert* Deletion Affects Body Weight and Protein Oxidation in the Heart

Over the entire duration of the experiment, SHR-*Tert*^−/−^ showed significantly lower body weight compared with SHR (2-way ANOVA; *P*=0.0001; for each age, *P*<0.0001; Figure S2A). The effect of *Tert* deletion on oxidative stress was tested in 5-month-old animals. In the serum, *Tert* deletion had no effect on the antioxidant power measured by Ferric Reducing Antioxidant Power (Figure S2B; n=18 per group, *t* test; *P*=0.8558). However, *Tert* deletion increased the level of protein oxidation in the left ventricle of the heart (Figure S2C; n=6 per group, *t* test; *P*=0.0248), as was proven by repeating the experiment twice. Representative membrane with detected carbonyl groups in protein side chains shows higher chemiluminescence intensity in SHR-*Tert*^−/−^ (Figure S2D). The integrated density of each band was normalized to the protein loading calculated from the Ponceau-stained membranes.^32^ Data were normalized against Ponceau S-stained membrane (Figure S2E).

### SHR-*Tert*^−/−^ Rats Are Less Active and Take More Rest Breaks During the Active Phase

**Figure 1.**
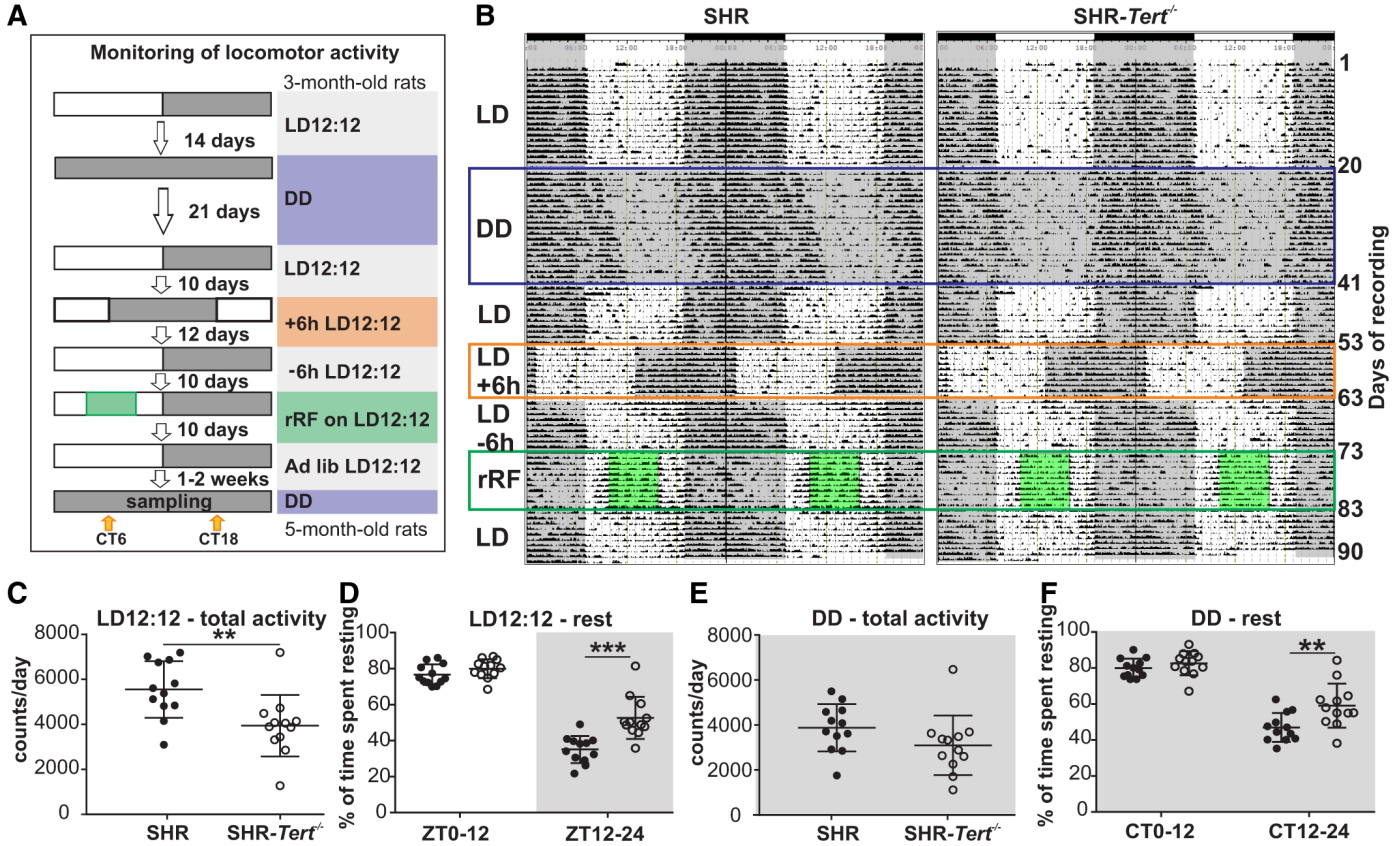
**Locomotor activity, but not its circadian regulation, is affected in spontaneously hypertensive rat (SHR)-*Tert^−/−^*. A**, Protocol of the locomotor activity recording. **B**, Representative activity records (actograms) of one SHR and one SHR-*Tert*^−/−^ obtained over a 90-day experimental protocol described in **A**. The actograms of all animals are shown in Figure S3. **C**, Total activity under standard light-dark regimen with 12 hours of light and 12 hours of darkness (LD12:12). **D**, Relative inactivity time on LD12:12. **E**, Total activity under constant darkness, or constant darkness (DD). **F**, Relative inactivity time on DD. Data presented on **C** through **F** were obtained during the first half of the experiment (upper part of **A**) when animals were 3 months (LD12:12 data) and 3.5 to 4 months (DD data) old. Activity fragmentation, period, adjustment to shifts in LD12:12, and feeding regime are shown in Figure S4. Data are presented as individual values with mean±SD, *t* test (**C** and **E**) or 2-way ANOVA (**D** and **F**). n=12 for each group. *P<0.05, **P<0.01, and ***P<0.001.

The protocol of activity recording is shown in Figure 1A. Locomotor activity started to be monitored in 3-month-old SHR-*Tert*^−/−^ (n=12) and SHR (n=12); the representative double-plotted activity recordings (actograms) of one SHR and one SHR-*Tert*^−/−^ with marked intervals for changes in the protocol are shown in Figure 1B (the actograms for all animals are shown in Figure S3).

In standard light-dark regimen with 12 hours of light and 12 hours of darkness (LD12:12), the total activity over a 24-hour interval (Figure 1C) was significantly lower in the SHR-*Tert*^−/−^ compared with the SHR (*t* test; *P*=0.007). Calculation of duration of inactivity (resting time; Figure 1D) showed that the SHR-*Tert*^−/−^ spent more time resting during the dark phase (zeitgeber time [ZT] 12–ZT24; *t* test; *P*=0.0002) but not during the light phase (ZT0–ZT12; *t* test; *P*=0.16) compared with the SHR. In addition, the locomotor activity evaluated in 5-minutes blocks throughout the 24-hour cycle was more fragmented in SHR-*Tert*^−/−^ (Figure S4A), as they had higher percentage of shorter activity bouts (2-way ANOVA; <30 minutes: *P*=0.0096; 35–60 minutes: *P*=0.0063) and a lower percentage of longer activity bouts (2-way ANOVA; >90 minutes: *P*<0.0001) compared with SHR. Consistent with this, SHR-*Tert*^−/−^ had a lower percentage of rest breaks shorter than 30 minutes over the 24-hour period (Figure S4B).

On constant darkness (DD), the total activity over the 24-hour interval was not significantly different in SHR-*Tert*^−/−^ compared with SHR (*t* test; *P*=0.12; Figure 1E), but the percentage of time they spent resting during the active phase of the circadian cycle (circadian time [CT] 12–CT24) was higher (*t* test; *P*=0.009; Figure 1F). In addition, the activity was more fragmented as the percentage of activity bouts shorter than 30 minutes was higher (2-way ANOVA; *P*=0.0130), and the percentage of activity bouts longer than 90 minutes was lower (2-way ANOVA; *P*=0.0001; Figure S4C). Fragmentation of resting time was not different between both groups (Figure S4D), suggesting no effect on sleep. Rats of both groups exhibited apparent resting interval in their locomotor activity during the second half of the subjective night, but the SHR-*Tert*^−/−^ rested more (*t* test; *P*=0.0006; Figure S4E) with longer continuous breaks (*t* test, *P*<0.0001; Figure S4F) compared with SHR.

### *Tert* Deletion Does Not Affect the Ability of the Central Clock to Regulate Locomotor Activity

Comparison of circadian parameters in DD between both groups showed that the period of locomotor activity did not differ (SHR: 24.09±0.06, n=12; SHR-*Tert*^−/−^: 24.11±0.06, n=12; *t* test: *P*=0.4325; Figure S4G). However, the duration of the free-running activity on the DD condition, when entraining signals are lacking, was prolonged due to the gradual advancement of the onset of activity. This resulted in a longer activity duration (α; *t* test; *P*=0.0127; Figure S4H). SHR-*Tert*^−/−^ maintained on LD12:12 were able to re-entrain to the shifts in the LD12:12 cycle similar to SHR (2-way ANOVA; *P*=0.8545; Figure S4I). The results indicate that the central clock of SHR-*Tert*^−/−^ is still able to drive circadian rhythms endogenously and entrain to the shifts in the external LD12:12 cycle. The *Tert* deletion had no effect on the reorganization of the activity pattern due to the reversal in the feeding regime. During the 10 days of the restricted feeding, animals of both groups gradually developed anticipatory activity during 2 hours before food was provided at ZT3 (2-way ANOVA; *P*=0.3811; Figure S4J), and the activity detected before (ZT0–ZT3), during (ZT3–ZT9), and after (ZT9–ZT12 and ZT12–ZT24) food presence did not differ between the groups (2-way ANOVA; *P*=0.0935; Figure S4K).

### *Tert* Deletion Abolishes Circadian Rhythm in BP in SHR

**Figure 2.**
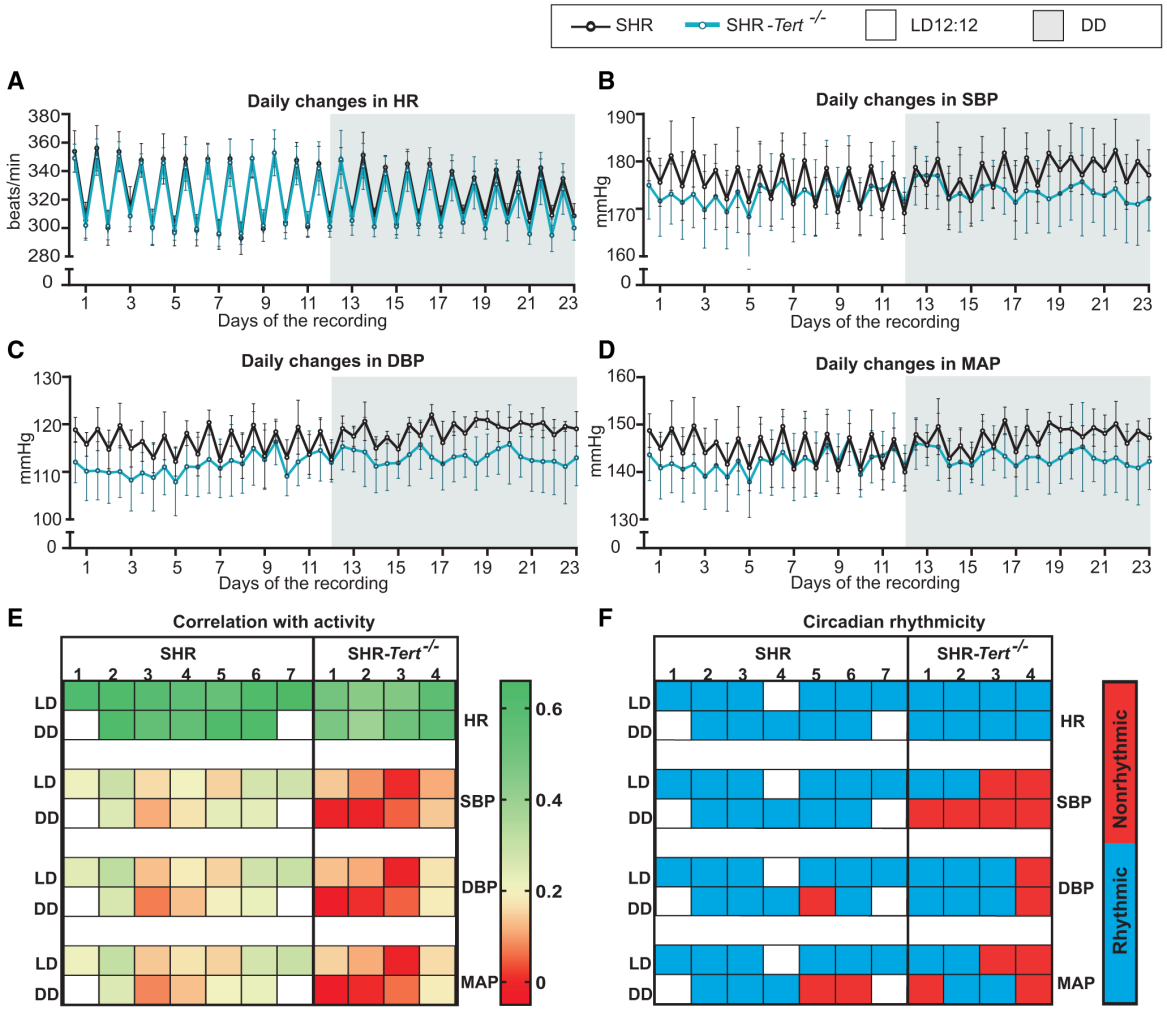
**Circadian regulation of blood pressure is affected in spontaneously hypertensive rat (SHR)-*Tert^−/−^*.** Profile of day and night variation of heart rate (HR; **A**), systolic blood pressure (SBP; **B**), diastolic blood pressure (DBP; **C**), and mean arterial pressure (MAP; **D**) monitored in standard light-dark regimen with 12 hours of light and 12 hours of darkness (LD12:12) for 12 days (white area) and then in constant darkness (DD) for 11 days (shaded area) in SHR (black) and SHR-*Tert*^−/−^ (blue). **E**, Schematic summary of statistical evaluation of circadian rhythmicity in individual SHR and SHR-*Tert*^−/−^ on LD12:12 and DD (detailed data shown in Table S3). Blue is for statistically significant, and red is for nonsignificant results. **F**, Heatmap showing the correlation of the cardiovascular parameters (HR, DBP, SBP, and MAP) with spontaneous activity of the animals. Red is the lowest value, and blue is the highest (detailed data shown in Figure S6). The open boxes are for missing data due to failure in telemetry recording or the death of one animal. In total, 7 SHR and 4 SHR-*Tert*^−/−^ 4-month-old animals were recorded.

Data for heart rate (HR), systolic BP (SBP), and diastolic BP (DBP) collected by telemetry (described in the study by Vaneckova et al^33^) were compared between rats of both groups maintained under LD12:12 for 12 days, which were followed by 11 days of DD (Figure 2). The presence or absence of the circadian rhythms in the measured parameters was performed using an open-source online platform BioDare2.^34^ Period, circadian phase, and amplitude were measured using the mFourfit model.^35^ Day/night dipping pattern was considered reversed if the difference was negative.^36^ The profiles of cumulative day/night and subjective day/subjective night data for the LD12:12 and DD recordings, respectively, are shown in Figure 2A for HR, Figure 2B for SBP, Figure 2C for DBP, and Figure 2D for mean arterial pressure (MAP).

HR in SHR-*Tert*^−/−^ maintained on LD12:12 and DD did not differ from SHR in the day/night amplitude (Figure S5A; 2-way ANOVA; *P*=0.9591 and *P*=0.3371, respectively), the mean HR during the active period (Figure S5B; 2-way ANOVA; *P*=0.7534), or the rest period (Figure S5C; *t* test; *P*=0.8419). In both SHR and SHR-*Tert*^−/−^, HR was 12% to 16% and 7% to 13% lower during the inactive phase in LD12:12 and DD, respectively (Figure S5E), with no differences between control and knockout animals (2-way ANOVA; *P*=0.5868). Also, the HR profile phase was not shifted in SHR-*Tert*^−/−^ compared with SHR (Figure S5D; 2-way ANOVA; *P*=0.9327). The presence of a circadian rhythm in HR (pBH < 0.001) was confirmed for all SHR and SHR-*Tert*^−/−^ maintained in LD12:12 and DD (Figure 2E; Table S3).

The SBP profile of SHR-*Tert*^−/−^ showed a significantly lower day/night amplitude compared with SHR in both LD12:12 and DD (Figure S5G; 2-way ANOVA; *P*=0.0174; post hoc *P*=0.0269 on both LD12:12 and DD). Mean SBP during the active phase (2-way ANOVA; *P*=0.1580; Figure S5H) or during the rest phase (2-way ANOVA; *P*=0.6422; Figure S5I) was not different. Both SHR and SHR-*Tert*^−/−^ showed a pathological day-night dipping pattern (<10%). On LD12:12, SBP decreased less in SHR-*Tert*^−/−^ than in SHR during the inactive phase (2-way ANOVA; *P*=0.003; post hoc *P*=0.0036). On DD, the same pattern was observed (post hoc *P*=0.0075), with 2 of 4 knockout animals reaching the point of the “reverse dipping” (difference below zero; Figure S5K). Similar to HR, the phase of the SBP profile was not shifted (2-way ANOVA; *P*=0.7437; Figure S5J). SBP exhibited a circadian rhythm (pBH<0.001) in all SHR rats maintained on both LD12:12 and DD, but, of the 4 SHR-*Tert*^−/−^, only 2 had the SBP rhythm on LD12:12, and all lost the rhythm on DD (pBH>0.001 in all SHR-*Tert*^−/−^ rats on DD; Figure 2E; Table S3).

The day/night amplitude of the DBP profile in SHR-*Tert*^−/−^ was significantly suppressed on LD12:12 and DD compared with SHR (Figure S5K; 2-way ANOVA; *P*=0.0066; post hoc *P*=0.0094 on LD12:12 and *P*=0.0053 on DD). In addition, the mean DBP was significantly lower during the active phase (Figure S5L; 2-way ANOVA; *P*=0.0213; post hoc *P*=0.0243 on both LD12:12 and DD) but not during the rest phase (Figure S5M; 2-way ANOVA; *P*=0.1743). The difference between day and night DBP values was no more than 10% for SHR and SHR-*Tert*^−/−^. On both LD12:12 and DD, DBP during the inactive phase decreased in SHR-Tert^−/−^ less than in SHR (2-way ANOVA; *P*=0.0099; post hoc *P*=0.0072 and *P*=0.038). One SHR-*Tert*^−/−^ animal was a reverse dipper on both light regimes (Figure S5Q). In addition, the phase of the DBP profile was significantly advanced in SHR-*Tert*^−/−^ (Figure S5N) (2-way ANOVA; *P*=0.0091; post hoc *P*=0.0234 on both LD12:12 and DD). The DBP exhibited circadian rhythm in SHR on LD12:12 and DD (pBH<0.001), except for one SHR on DD (pBH=0.1569). In the SHR-*Tert*^−/−^ group, the DBP rhythm was present in 3 animals on both LD12:12 and DD (pBH<0.001) and was lost in one SHR-*Tert*^−/−^ regardless of the light condition (pBH=0.00232 on LD12:12; pBH=0.00218 on DD; Figure 2E; Table S3).

The MAP profile of SHR-*Tert*^−/−^ showed a significantly lower day/night amplitude compared with SHR in both LD12:12 and DD (Figure S5S; 2-way ANOVA; *P*=0.007; post hoc *P*=0.0092 on both LD12:12 and DD). Mean MAP during the active phase (2-way ANOVA; *P*=0.1145; Figure S5T) or during the rest phase (2-way ANOVA; *P*=0.4909; Figure S5U) was not different. Both SHR and SHR-*Tert*^−/−^ exhibited a pathological day-night dipping pattern (<10%). On LD12:12, MAP decreased less in SHR-*Tert*^−/−^ than in SHR during the inactive phase (2-way ANOVA; *P*=0.0081; post hoc *P*=0.0074). On DD, the same pattern was observed (post hoc *P*=0.0309), with little to no dipping in SHR-*Tert*^−/−^ (Figure S5W). Similar to DBP, the phase of the MAP profile was significantly advanced (2-way ANOVA; *P*=0.054; Figure S5V). On LD12:12, MAP exhibited a circadian rhythm (pBH<0.001) in all SHR and in 2 SHR-*Tert*^−/−^. On DD, 2 SHR and 2 SHR-*Tert*^−/−^ show no rhythmicity in MAP (Figure 2E; Table S3).

Correlation matrices (Spearman correlation) generated in Python for all parameters measured by telemetry (HR, SBP, DBP, MAP, pulse pressure, temperature, and activity) in SHR and SHR-*Tert*^−/−^ maintained under LD12:12 and DD are shown in Figure S6. The correlations of the actual activity state with HR (Figure S5F; 2-way ANOVA; *P*=0.0038; post hoc *P*=0.0059 on both LD12:12 and DD), SBP (Figure S5L; 2-way ANOVA; *P*=0.0010; post hoc *P*=0.0026 on both LD12:12 and DD), DBP (Figure S5R; 2-way ANOVA; *P*=0.0158; post hoc *P*=0.0401 on both LD12:12 and DD), and MAP (Figure S5X; 2-way ANOVA; *P*=0.0153; post hoc *P*=0.0441 on both LD12:12 and DD) were significantly weaker in SHR-*Tert*^−/−^ compared with SHR (Figure 2F).

Overall, the results show that in SHR-*Tert*^−/−^, both SBP and DBP exhibit smaller differences between the active and resting phases (amplitude) compared with SHR, which can be attributed to their lower values during the active phase at both LD12:12 and DD. DBP in SHR-*Tert*^−/−^ showed lower mean daily levels both on LD12:12 and DD. A smaller difference between the BP values during the rest and activity period also contributes to the abnormal/reversed day/night dipping patterns that are more pronounced in DD. Importantly, the circadian rhythm of SBP was completely abolished in all SHR-*Tert*^−/−^ under DD conditions.

### The Number of Tyrosine Hydroxylase-Positive Cells in the RVLM Is Reduced in SHR-*Tert*^−/−^

**Figure 3.**
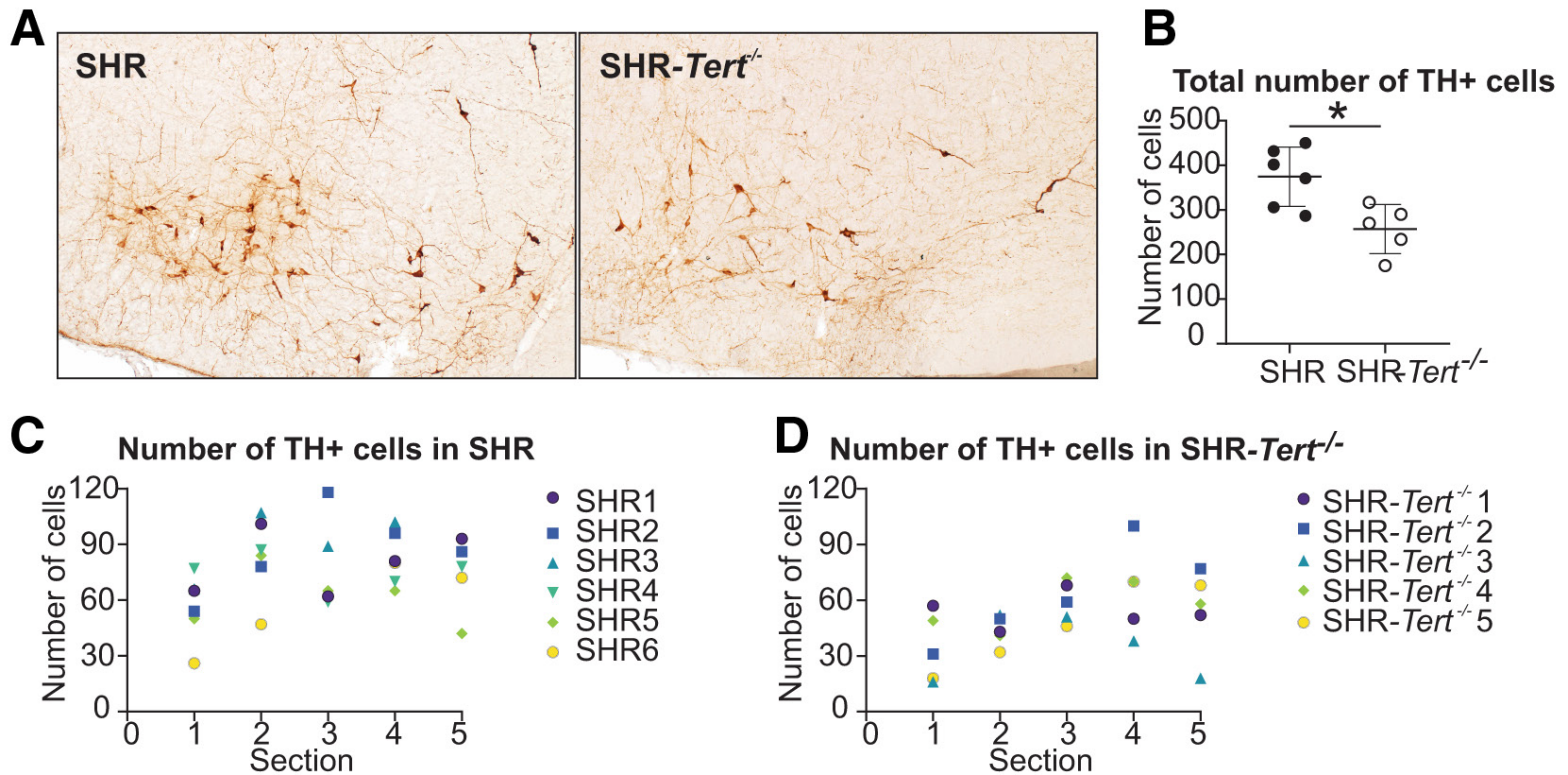
**Number of TH (tyrosine hydroxylase)-immunopositive cells in the blood pressure regulating center, rostral ventrolateral medulla (RVLM). A**, Representative brain section with RVLM of a 5-month-old spontaneously hypertensive rat (SHR) and SHR-*Tert*^−/−^. **B**, Total number of TH-positive cells was significantly lower in SHR-*Tert*^−/−^. Cells were counted on 5 sections per brain of SHR (**C**) and SHR-*Tert*^−/−^ (**D**). Data are presented as individual values with mean±SD, *t* test. n=6 for SHR; n=5 for SHR-*Tert*^−/−^. * *P*<0.05, **P<0.01, and ***P<0.001.

To determine the mechanism of disrupted circadian regulation of BP, we used immunohistochemistry^37^ for comparison of the number of adrenergic (TH [tyrosine hydroxylase]-immunopositive) cells in the RVLM of SHR (n=6) and SHR-*Tert*^−/−^ (n=5; Figure 3). We followed the localization of the adrenergic cells in the medulla, as described in previous studies.^38^ We used TH as a marker of adrenergic cells because (1) TH catalyzes the first step of adrenaline synthesis from L-tyrosine^39^ and (2) most cells containing TH in RVLM have been shown to also contain all other enzymes necessary for adrenaline production.^40^ Representative sections are shown in Figure 3A. The total number of cells (Figure 3B) counted in 5 consecutive sections through the RVLM (Figure 3C and 3D) was significantly lower in SHR-*Tert*^−/−^ compared with SHR (*t* test; *P*=0.0118).

### *Tert* Deletion Significantly Affects Circadian Clocks in the Heart Atrium and Other Peripheral Tissues

**Figure 4.**
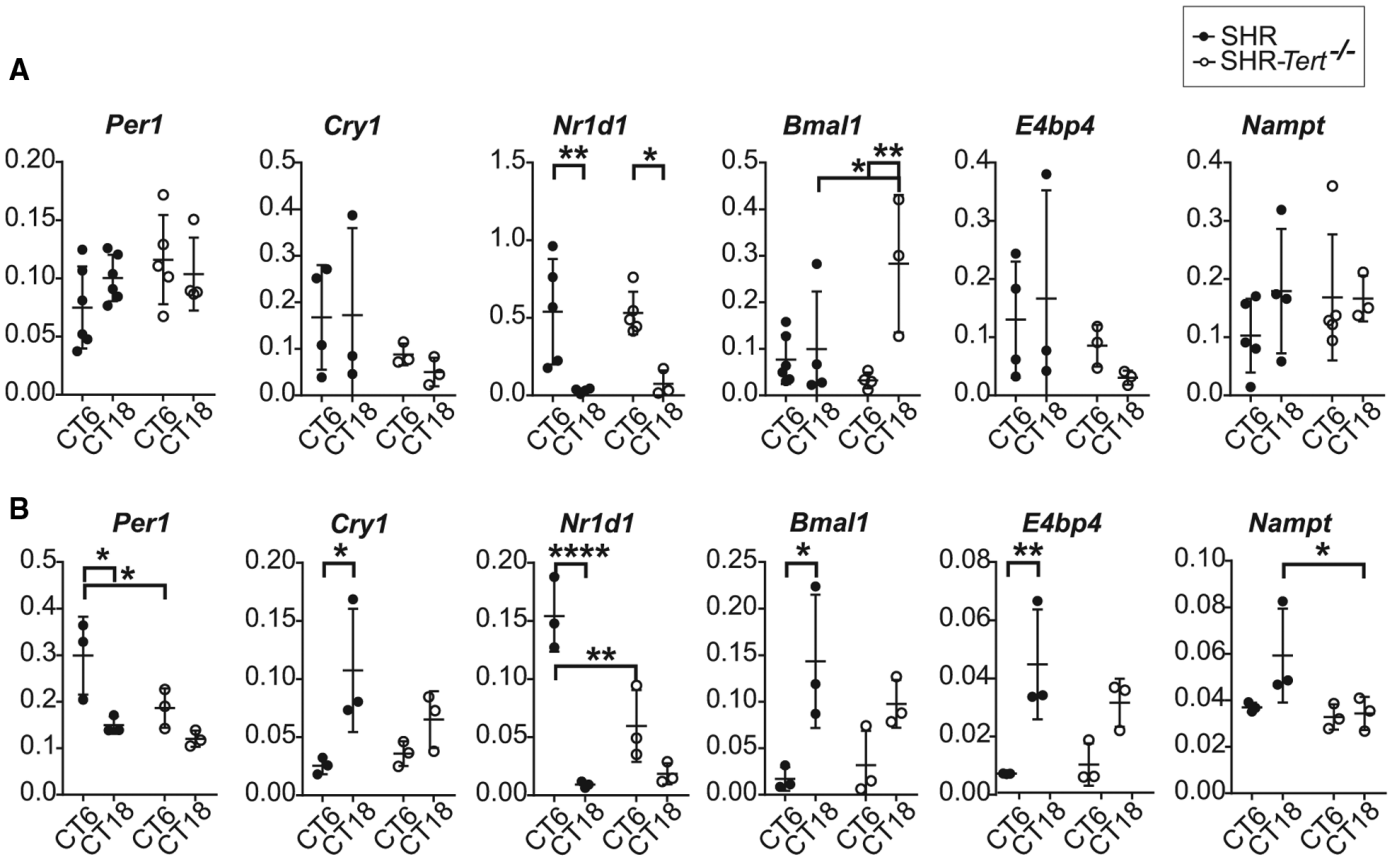
**Expression of clock genes in the heart is affected in spontaneously hypertensive rat (SHR)-*Tert^−/−^*.** Expression of 6 genes (*Per1*, *Cry1*, *Nr1d1*, *Bmal1*, *E4bp4*, and *Nampt*) was compared in the heart atrium collected from 5-month-old SHR and SHR-*Tert*^−/−^ at subjective day (circadian time [CT] 6) and subjective night (CT18; **A**) on day 1 (n=3–6 per each group and time point) and (**B**) on the 14th day in constant darkness (DD; n=3 per each group). For statistics, see Table S4. Data are presented as individual values with mean±SD, 2-way ANOVA. *P<0.05, **P<0.01, and ***P<0.001.

We used reverse transcription PCR (RT-PCR; list of primes is provided in Table S2) analyzed by the Livak ΔΔCt method^41^ to detect expression of 5 clock genes (*Per1*, *Per2*, *Cry1*, *Nr1d1*, and *Bmal1*), 3 clock- and metabolism-related genes (*E4bp4*, *Dbp*, and *Nampt*) in right atrium-ATR (Figure 4), and 3 other peripheral tissues (gonadal white adipose tissue-gWAT), liver-LIV, pancreas-PAN; Figure S7) collected from SHR and SHR-*Tert*^−/−^ at subjective day (CT6) and subjective night (CT18) on day 1 (Figure 4A; Figure S7) and the 14th day in DD (Figure 4B; Figure S7). In the SHR controls, the differences in gene expression levels between CT6 and CT18 were gene-specific, corresponding to the tissue-specific phases of their circadian profiles determined by the operational transcription-translation feedback loop. Deletion of *Tert* affected the differences between the expression of individual genes at CT6 and CT18 (for statistics, see Table S4).

In the atrium on day 1 of DD (Figure 4A), the difference between *Bmal1* mRNA levels at CT6 and CT18 in SHR-*Tert*^−/−^ lost statistical significance, which was due to significantly higher levels in SHR-*Tert*^−/−^ compared with SHR at CT18 (*P*=0.0376). After 14 days in DD (Figure 4B), the effect of *Tert* deletion was most pronounced because the expression of all genes in SHR-*Tert*^−/−^ lost the significant difference between CT6 and CT18.

In the liver on day 1 of DD (Figure S7), the significant differences between CT6 and CT18 in SHR-*Tert*^−/−^ were abolished for *Per1* and *Per2* and, in opposite, were significant for *Dbp*. After 14 days in DD (Figure S7), the difference between CT6 and CT18 was lost in SHR-*Tert*^−/−^ for *Per2* and *Cry1* (the level at CT18 was significantly lower in SHR-*Tert*^−/−^; *P*=0.0360) and *Bmal1* and *E4bp4* (the level at CT18 was significantly lower in SHR-*Tert*^−/−^; *P*=0.0422).

In the gWAT on day 1 of DD (Figure S7), the differences between CT6 and CT18 were completely abolished for *E4bp4* (the level at CT18 was significantly lower in SHR-*Tert*^−/−^; *P*=0.0321) and *Dbp*. In contrast to SHR, the difference was significant for *Bmal1* in SHR-*Tert*^−/−^. After 14 days in DD (Figure S7), the difference between CT6 and CT18 for *Cry1* was reduced due to a significantly higher level at CT6 in SHR-*Tert*^−/−^ (*P*=0.0116) and completely abolished for *Nr1d1*, *Bmal1*, and *E4bp4*.

In the pancreas on day 1 of DD, the difference between CT6 and CT18 for *Dbp* was reduced in SHR-*Tert*^−/−^ due to significantly lower levels at CT6 (*P*=0.0308). The difference was significant for *Cry1* in SHR-*Tert*^−/−^ but not in SHR. After 14 days in DD (Figure S7), the difference was abolished for *Cry1* and *Nr1d1* (the levels in SHR-*Tert*^−/−^were significantly lower at CT6; *P*=0.0312).

The expression levels of genes not mentioned in the individual tissues did not differ between CT6 and C18 or between SHR and SHR-*Tert*^−/−^. Overall, deletion of *Tert* affected peripheral circadian clocks, resulting in a reduction or abolition of subjective day/night differences in the expression of several genes immediately after the animals were placed on DD, and the effect was even stronger after 14 days on DD. Noteworthy, the atrium was the most affected tissue.

## Discussion

To investigate the effects of *Tert* deletion on the circadian regulation of cardiovascular function, we created an SHR-*Tert*^−/−^ model whose F3 generation has shortened telomeres in the spleen, liver, heart, kidney, and intestine. From 3 months of age, the F3 SHR-*Tert*^−/−^ exhibited signs of senescence at the systemic level, as they (1) had lower body weight and lower activity fitness (lower level, more fragmented with longer breaks, and more scattered activity patterns) and (2) exhibited oxidative stress in the heart. While circadian regulation of behavioral activity was preserved in SHR-*Tert*^−/−^, circadian regulation of cardiovascular function was severely impaired as documented by a reduced day/night variation in SBP and DBP levels in animals maintained under LD12:12 and DD, and a complete loss of circadian rhythm in SBP in DD. Our data suggest that the effect may be mediated via disruption of rhythmic signaling from the brain regulatory regions to the periphery, as we have shown that in SHR-*Tert*^−/−^ (1) the number of TH-immunopositive cells in the RVLM of the brainstem is significantly reduced and (2) day/night changes in levels of clock gene expression in the heart (and other tissues) are affected.

Disturbances in both circadian and cardiovascular regulations are major hallmarks of aging. In the elderly, cardiometabolic disorders develop in humans due to predisposition or due to an unfavorable lifestyle. To investigate the mechanism, we examined the effect of the *Tert* deletion on the circadian regulation of BP in an animal model that, over the lifespan, spontaneously develops essential hypertension^42^ and other metabolic-related pathology.^43,44^ We have previously shown that SHR has an aberrant circadian system as the central clock and its output rhythm in locomotor activity are phase-advanced compared with control normotensive rats.^45^ Also, expression of clock genes in the peripheral tissues examined in this study, such as heart,^46^ liver,^47^ fat,^48^ and pancreas,^49^ is modulated by the secretion of 2 hormones that regulate BP, namely, corticosterone and aldosterone, which are phase shifted in SHR.^50^ In addition, SHR is more sensitive to the temporal reversal of the feeding regimen and responds more robustly both behaviorally and at the level of clock gene expression in the peripheral tissues.^47^ Although a causal link between the pathology and the disrupted circadian function in SHR has not been established, results from previous studies suggest that the hypertensive phenotype can be partially restored by introducing rhythmic conditions. For example, restricting the access to food only to the dark phase of LD12:12 improved rhythm in BP, as well as clock- and metabolism-related gene expression in cardiovascular tissues.^48^ In addition, regular rhythmic maternal care provided to SHR pups by a normotensive foster mother prevented them from the development of the spontaneous rise in HR at 2 months of age.^51^ Furthermore, it has been shown that changes in circadian regulation of locomotor activity precede the development of spontaneous hypertension in the SHR,^52^ further supporting causality between aberrant circadian regulation and the development of hypertension. Thus, the SHR serves as a model that spontaneously develops hypertension and has a close relationship with a higher sensitivity to circadian dysregulation.^53^ Therefore, we selected SHR as a background strain relevant to the clinical context of elderly patients with cardiovascular disease.

We confirmed that *Tert* deletion leads to telomere shortening in SHR-*Tert*^−/−^, which is associated with some of the specific attributes of aging. Indeed, SHR-*Tert*^−/−^ exhibited signs of age-related cachexia. Compared with age-matched SHR, the SHR-*Tert*^−/−^ had significantly lower body weight and reduced physical fitness as evidenced by lower spontaneous locomotor activity measured by IR detectors in the cage. The activity was reduced during their active phase when they took a longer break (siesta) in its second half and was more fragmented over the 24-hour interval, be it in LD12:12 or DD conditions.

Oxidative stress is another hallmark that causally links impaired circadian regulation, hypertension, and aging. It was previously shown that cardiac stem cells of SHR exhibit higher oxidative stress than those of normotensive Wistar rats.^7^ Our results suggest that deletion of *Tert* facilitates this process, as SHR-*Tert*^−/−^ exhibited a higher level of oxidative stress in the heart compared with control SHR. Nevertheless, further investigations are required, as the assessment of oxidative stress is limited to protein carbonylation (detected by OxyBlot), which reflects only one aspect of oxidative damage.

Both pathological states (oxidative stress and cachexia) have been associated with disrupted circadian regulation.^54,55^ Therefore, we first tested the effect of *Tert* deletion on the function of the central clock in the SCN using the parameters of the output rhythm in locomotor activity in animals exposed to DD (to detect the endogenous period) and 6-hour phase shifts in LD12:12 (to detect the phase-shifting ability). The results show that *Tert* deletion and telomere shortening have no effect on the functionality of the central clock in the SCN to drive and entrain this output rhythm. However, we found that *Tert* deletion had a significant effect on circadian regulation of BP in SHR-*Tert*^−/−^. Indeed, the amplitudes of daily profiles in SBP and DBP were suppressed in LD12:12 and DD due to a lower level of BP during the time of day when the rats were active. Importantly, our data show a weaker correlation between the level of activity and SBP, DBP, and HR in SHR-*Tert*^−/−^ compared with SHR, suggesting that the lower level of BP in SHR-*Tert*^−/−^ is not solely due to their lower activity. In addition, the circadian rhythm in SBP was completely abolished in DD. Such an effect of *Tert* deletion on the circadian rhythm of cardiovascular function has not yet been described although the causal relationship between telomere activity and hypertensive phenotype has been extensively studied.^56^ A previous study found that F1 and F3 generations of TERC-deficient mice (normotensive background) develop hypertension.^57^ However, in animals with hypertension (SHR), the role of telomerase activity in the development of hypertension seems rather controversial.^56^ We found that despite telomere shortening, our SHR-*Tert*^−/−^ had significantly lower mean levels of DBP, which does not support its causal role of telomere shortening in the hypertension development. Our data are consistent with the results of a human study, showing that patients with hypertension with lower blood TERT concentration had ineffective BP control and worse metabolic profiles in adipose tissues.^58^

To determine the mechanism of disrupted BP rhythmicity in SHR-*Tert*^−/−^, we attempted to assess the effect of *Tert* deletion on the regulation of BP at the level of the brainstem regulatory region RVLM. We found that SHR-*Tert*^−/−^ has a lower number of TH-positive cells in the RVLM, suggesting a lower sympathetic outflow to the peripheral clocks. Therefore, we assessed actual states of the peripheral circadian clocks, namely, in the heart atrium, liver, gWAT, and pancreas, by analyzing the expression of clock genes at the tissue level. To accomplish this task and to cope with the limited availability of the mutant animals, we adopted an approach to determine the expression levels of multiple clock and clock-related genes at 2 time points, that is, subjective day (CT6) and subjective night (CT18). Because these genes are expressed rhythmically with phases distributed over 24 hours according to the transcription-translation feedback loop, a change in gene-specific ratios indicated a modulation of their complete diurnal profiles and allowed a comparison of the effects between SHR and SHR-*Tert*^−/−^. We found that the effect of *Tert* deletion on circadian clocks is tissue-specific. The results suggest an effect of *Tert* deletion on desynchronization rather than dampening of the oscillators, as the differences in CT6/CT18 mRNA levels were not abolished for all genes, but rather altered for some of them. The most significant effect of *Tert* deletion was seen in the heart atrium, where the statistical significance of the difference between the day/night levels was abolished for almost all genes after exposure to DD, consistent with the loss of the circadian rhythm in BP.

### Limitations

Our study was not designed to distinguish between the role of telomere shortening and *Tert* deletion in the disruption of circadian BP regulation or their combined effects. *Tert* has been shown to have noncanonical effects that may influence physiology beyond telomere maintenance.^59^ Namely, the effect of *Tert* on the regulation of transcription of specific genes^60^ and protection against oxidative stress and mitochondrial dysfunction^61^ are potential mechanisms that are independent of telomere shortening. Another limitation of our study is the lower number of SHR-*Tert*^−/−^ animals used for the telemetry measurements, as they had a lower survival rate after the surgery required to place the telemetry sensors. This could be explained by higher inflammation and oxidative stress in SHR-*Tert*^−/−^ compared with SHR. This study did not focus on the effects of deletion of *Tert* on the circadian clock in the kidney and brain regions, which will be further investigated in future studies. Finally, this study did not focus on the interaction between the *Tert* and clock genes on a molecular basis.

### Perspectives

The mechanisms of the age-dependent deterioration in the circadian regulation of physiological functions have not yet been clarified. The results of this study provide the first evidence for a *Tert* knockout-induced impairment of circadian regulation of the BP, which represents a possible mechanism underlying the age-related impairment of the regulation of this physiological function. Our results show that deletion of the *Tert* gene in animals that spontaneously develop hypertension not only leads to impaired fitness but, more importantly, abolishes circadian rhythm in BP. We show that the mechanism of the latter is mediated downstream of the central clock in the SCN and is likely related to a reduced number of adrenergic cells in the RVLM, a region involved in regulating adrenergic innervation of the periphery, leading to poor synchrony of peripheral circadian oscillators in the heart (and other tissues). Taken together, these data provide evidence for a new possible mechanism for the deterioration of the temporal regulation of physiological functions, including the diurnal rhythm of BP, in the elderly.

## ARTICLE INFORMATION

### Acknowledgments

The authors declare no assistance from any artificial intelligence technologies.

### Author Contributions

K. Semenovykh was involved in collection of samples, analysis of telemetry data, immunohistochemistry, OxyBlot, Ferric Reducing Antioxidant Power, figures, statistics, and draft of the manuscript. M. Pravenec was involved in generation of the animal model, figures, draft of the manuscript, and funding acquisition. I. Vaněčková was involved in surgery and telemetry recording, and manuscript revision. P. Houdek was involved in collection of samples, RNA isolations, locomotor activity monitoring and analysis, feeding regime, immunohistochemistry, figures, statistics, and draft of the manuscript. M. Sládek was involved in RT-qPCR of samples from peripheral tissues, figures, statistics, and draft of the manuscript. M. Šimáková was involved in the genotyping of spontaneously hypertensive rat (SHR)-*Tert*^−/−^ rats. P. Mlejnek was involved in genotyping and sequencing of SHR-*Tert*^−/−^ founders. S. Selvi was involved in collection of samples, RNA isolations, RT-qPCR of peripheral tissues, figures, and statistics. J. Šilhavý was involved in generation of SHR-*Tert*^−/−^ founders. F. Liška was involved in the design of the ZFN-targeting sequence, measurement of telomere length, and figures. D. Semenovykh was involved in RNA isolation of heart samples and OxyBlot. S. Hojná was involved in surgery and telemetry recording.

A. Sumová was involved in conceptualization, supervision, data analysis and interpretation, figures, draft of the manuscript, and final version of the manuscript.

### Sources of Funding

The project no. LX22NPO5104 is funded by the European Union Next Generation EU and the Research Project RVO: 67985823. M. Pravenec was supported by grant LUAUS23095 within the INTER-EXCELLENCE Program of the Ministry of Education, Youth, and Sports of the Czech Republic

### Disclosures

None.

### Supplemental Material

Supplemental Materials and Methods

Tables S1–S4

Figures S1–S7

Data File S1

## Supplementary Material


