# Supplementary material for: Tert Deletion Impairs Circadian Regulation of Blood Pressure in Male Spontaneously Hypertensive Rats
Source: Hypertension. 2025 Dec 10;83(2):e25510. doi: 10.1161/HYPERTENSIONAHA.125.25510 (PMC12822765; doi:10.1161/HYPERTENSIONAHA.125.25510)
Supplement: Supplementary file 1 [file hyp-83-e25510-s001.pdf]

Supplemental Materials

Tert deletion impairs circadian regulation of blood pressure in male spontaneously hypertensive rats

Kateryna Semenovykh[1], Michal Pravenec[2,3], Ivana Vaněčková[4], Pavel Houdek[1], Martin Sládek[1], Miroslava Šimáková[2], Petr Mlejnek[2], Saba Selvi[1], Jan Šilhavý[2], František Liška[2,3], Dmytro Semenovykh[1], Silvie Hojná[4], Alena Sumová[1*]

[1]*Laboratory of Biological Rhythms, Institute of Physiology, Czech Academy of Sciences, Prague, Czech Republic*
[2]*Laboratory of Genetics of Model Diseases, Institute of Physiology, Czech Academy of Sciences. Prague, Czech Republic*
[3]*Institute of Biology and Medical Genetics, First Faculty of Medicine, Charles University and General University Hospital, 12800 Prague, Czech Republic*
[4]*Laboratory of Experimental Hypertension, Institute of Physiology, Czech Academy of Sciences, Prague, Czech Republic*

*****Address for correspondence: Prof. PharmDr. Alena Sumová, DSc., Institute of Physiology of the Czech Academy of Sciences, Videnska 1083, 14200 Prague, Czech Republic, Tel. No: +420241062528, Fax No: +420241062488, Email: alena.sumova@fgu.cas.cz (ORCID: 0000-0003-4126-5470)

Short title: *Tert* KO impairs circadian rhythm in cardiac function

Key words: *Tert* knockout, rat, circadian clock, oxidative stress, blood pressure, heart rate

DETAILED MATERIALS AND METHODS

Animals

Spontaneously hypertensive rats SHR/OlaIpcv (referred to as SHR) from the breeding colony at the Institute of Physiology of the Czech Academy of Sciences were used to generate rats with homozygous *Tert* deletion (referred to as SHR-*Tert*$^{-/-}$) of the F3 generation (see below). Adult (2.5 to 5 months of age) male SHR and SHR-*Tert*$^{-/-}$ of F3 generation were used in the study. They were maintained under standard light/dark LD regimen with 12 h of light and 12 h of darkness (LD12:12). Light was turned on at 06.00 h (Zeitgeber time 0, ZT0) and turned off at 18.00 h (ZT12), and food and drinking water were available ad libitum throughout the experiment. These conditions changed during the experimental protocol to assess circadian parameters at the locomotor activity and gene expression level (as described below). In constant darkness (DD), the lights were not switched on, and the circadian time (CT) was defined according to the beginning of the inactive part (CT0) and the beginning of active part (CT12).

Ethical approval. The experimental protocol was approved by the Animal Care and Use Committee of the Institute of Physiology and complied with the Animal Protection Law of the Czech Republic as well as the European Community Council Directive 2010/63/EU.

Generation of Tert knockout SHR rats

The SHR-*Tert*$^{-/-}$ rats were obtained by microinjections of fertilized ova with Zinc Finger nuclease (ZFN) construct (Sigma Aldrich) by targeting *Tert* exon 1. ZFN binding site was 5´ GGCCGCTGGCGACCTTTG 3´ and 5´ GCCTGGGGCTTGAGG 3´ (upstream and downstream, respectively, corresponding to the +DNA strand).

For gDNA sequencing, the target site was amplified by 5´ TTTTCTAAGCACACCCCTGC 3´ and 5´ CTTGACATGTGAGCTAGGCG 3´ primers, and the purified PCR products (reference size 365 bp) were directly sequenced using BigDye1.1 Cycle Sequencing Kit (Thermofisher). The same PCR reaction was used for genotyping, with subsequent 10% acrylamide electrophoresis to detect fragment size. For routine genotyping, we also used shorter amplicons generated with primers 5´ CAAGCCGACTGCCCTCAA 3´ and 5´ CCGTGCGCTCTCTACTGC 3´ (reference size 90bp), and electrophoresed in 3.5% agarose gels.

For RNA sequencing, total RNA was extracted from tissue samples flash frozen in liquid nitrogen, stored at -80°C using RNeasy Plus Mini Kit (Qiagen) until lately reversely transcribed using SuperScript IV (Thermofisher). *Tert* was amplified with Phu DNA polymerase (New England Biolabs) with primers Tert_c5uF:5´ CCTGGTCAGTAGTCCCAAGC 3´ and Tert_c3uR: 5´ GGAGGACGACCTAGCTTCAC 3´. PCR products were sequenced using next-generation sequencing. Sequencing data were mapped by BWA-MEM2 (https://github.com/lh3/bwa) to *Tert* mRNA reference (NM_053423.2) in the Galaxy (https://galaxyproject.org and visualized using IGV (https://igv.org/).

Mutant rats were bred as heterozygotes by crossing with the SHR strain. Homozygous SHR-*Tert*$^{-/-}$ F1 generation was obtained by intercrossing SHR-*Tert*$^{+/-}$ heterozygotes. The *Tert* deletion was fixed by selecting *Tert*$^{-/-}$ breeding pairs from the intercross offspring. The subsequent F2 and F3 generations were obtained by mating F1 and F2 sibling rats, respectively. Rats from the F3 generation (used in this study) were infertile, so it was not possible to obtain F4 and subsequent generations.

Telomere Length Measurement

We measured relative telomere size using quantitative PCR. We selected albumin as a single copy gene control (primers Alb_c885F: 5´ GAACCAGGCCACTATCTCCA 3´ and Alb_c992R: 5´ AGATCGGCAGGAATGTTGTC 3´, respectively, 300 nM concentration in PCR reactions). 40 ng of genomic DNA (10 ng/ul) was input into 12 µL PCR reaction. Amplification reactions were performed in the Applied Biosystems 7900HT thermal cycler, using PowerUp SYBRGreen master mix (ThermoFisher Scientific). Relative telomere size was calculated using the $2^{-\Delta\Delta Ct}$ method (wild-type average = 1). Relative telomere size data was compared between wild-type SHR and SHR-*Tert*$^{-/-}$ animals using Cumming estimation plot method, https://www.estimationstats.com.

Serum Ferric Reducing Antioxidant Power (FRAP)

Blood collected by cardiac puncture was centrifuged at 4°C to obtain serum. Samples were stored in aliquots at -80°C until detection of FRAP using a FRAP assay kit (Merck). Briefly, 10 µL of the sample was combined with the reaction mix according to the manufacturer‘s instruction. After 1 h incubation at 37°C, endpoint absorbance was measured at 594 nm. Background was subtracted, and calculations were performed by interpolating unknown values from the standard curve.

Measurement of oxidative stress in the heart (OxyBlot assay)

The apical part of the left ventricle from each rat was frozen on dry ice and stored at -80°C until the assay. The 300 µl of the RIPA buffer supplemented with protease inhibitor (SigmaFast Protease Inhibitor Cocktail) were added to the samples, homogenized, and left on ice for 2 h for sufficient tissue lysis with a subsequent centrifugation at 12 000g for 3 minutes. The supernatant was collected, and protein concentration was measured by Bradford protein assay. Obtained samples were processed according to the manufacturer's instructions using Protein Oxidation Detection Kit (Sigma, S7150). Briefly, sample lysates underwent derivatization by 15 min incubation with DNPH solution or Derivatization-Control solution for control samples. Electrophoresis was performed on the Bolt Mini Gel Electrophoresis System (ThermoFisher) at 200 V with MOPS buffer (ThermoFisher). Roughly 5 µg of total protein was loaded per lane. Precision Plus Protein Dual Color Standards (Bio-Rad) standard was used as a molecular weight marker. Samples were then transferred from the gel to a nitrocellulose membrane using the Trans-Blot Turbo Transfer System (Bio-Rad). Membranes were washed in Tris-buffered saline with Tween 20 (TBST) and stained with Ponceau dye (Sigma Aldrich, P7170) for 5 minutes with gentle shaking. The membranes were scanned using the ChemiDoc System (Biorad, USA). The images obtained were used for further normalization. Membranes were blocked for 1 h at room temperature (RT) and incubated with primary antibodies from the OxyBlot kit overnight at 4 °C. On the second day, membranes were washed in TBST and incubated with a secondary antibody from the kit for 1 h at RT. After washing, membranes were developed by SuperSignal West Femto chemiluminescence substrate and scanned by ChemiDoc. Images were analyzed utilizing ImageLab 6.0 software (Biorad). The integrated density of each band was normalized to the protein loading calculated from the Ponceau-stained membranes. The signal from the membrane containing the negative control samples was subtracted prior to statistical analysis. The assay was repeated twice for each set of samples.

Monitoring of locomotor activity

To monitor the locomotor activity, the cages were equipped with infrared motion detectors positioned centrally over the top of each cage. The activity was recorded every minute using a circadian activity monitoring system (Dr. H.M. Cooper, INSERM, France),

and double-plotted actograms were generated to visualize the data. The parameters of circadian rhythmicity were analyzed using the ClockLab toolbox (Actimetrics, USA). The activity fragmentation was calculated individually for each animal as a percentage of the activity bouts longer than 5 min during the active part (i.e., ZT/CT12 – ZT/CT24) or inactive part of the cycle (ZT/CT0 – ZT/CT12).

Monitoring protocol. Individually housed rats (n = 12 per group) were enrolled into the experiment at 3 months of age when their locomotor activity started to be monitored over the entire protocol. The protocol included changes in external conditions as follows: LD12:12 for at least 2 weeks → constant darkness (DD) for 3 weeks → return to the original LD12:12 for 10 days → 6 h advance in LD12:12 for 12 days → return to the original LD12:12 for 10 days → restricted access to food for 6 h during the light phase of the LD12:12 (ZT3 – ZT9) for 10 days → return back to LD12:12 with ad libitum feeding for 1 to 2 weeks.

Detection of gene expression

Collection of samples. After completion of the protocol for monitoring of behavioral activity, the animals (age of 5 months; SHR: n = 12 and SHR-*Tert*$^{-/-}$: n = 12) were deeply anesthetized with pentobarbital sodium (50 mg/kg i.p.), weighed, and sacrificed with decapitation on the first day in the darkness at CT6 (SHR, n = 6; SHR-*Tert*$^{-/-}$, n = 6) and CT18 (SHR, n = 6; SHR-*Tert*$^{-/-}$, n = 6). Liver samples were collected in RNA-later buffer (Sigma), pancreas and heart right atrium were immediately homogenized in RLT buffer (Qiagen), and gonadal adipose tissue was frozen on dry ice. All samples were stored at -80°C until RNA isolation.
The other groups of animals (n = 6 per group) were sacrificed in DD after telemetry monitoring at circadian time CT6 (SHR, n = 3; SHR-*Tert*$^{-/-}$, n = 3) and CT18 (SHR, n = 3 SHR-*Tert*$^{-/-}$, n = 3). Peripheral tissues were removed and processed in the same manner before the animals were perfused for immunohistochemistry of brain sections (see below).

RNA isolation and real-time qPCR. Total RNA was isolated using RNeasy Mini Kit (Qiagen), and the final concentration was measured using the NanoDrop (ThermoFisher). RNA was then reverse transcribed into cDNA using the High-Capacity cDNA Reverse Transcription Kit (ThermoFisher) according to the manufacturer's protocol and diluted. The cDNA samples were analyzed by RT qPCR using DBdirect PCR SYBR Green Super Sense kit (Diana Biotechnologies, Czechia) with predesigned KicqStart primers (Sigma). Relative quantity of rat genes *Per1*, *Per2*, *Cry1*, *Nr1d1*, *Bmal1*, *E4bp4*, *Dbp*, and *Nampt* was quantified using Livak's ΔΔCt method against the geometric mean of three reference genes (*B2m, Gapdh, Tbp*). Sequences of the primers are shown in Supplementary Table S1. Data are expressed as the mean ± S.D.

Immunohistochemistry

SHR (n = 6) and SHR-*Tert*$^{-/-}$ (n = 5) maintained on DD for one week were deeply anesthetized with pentobarbital sodium (50 mg/kg, i.p.) and weighed. Perfusion was performed through the ascending aorta with the heparinized saline followed by PBS (0,01M sodium phosphate, 0.15 M NaCl, pH 7.2), and then freshly prepared 4% paraformaldehyde in PBS. Brains were removed, postfixed for 24 h at 4° C, and cryoprotected in 20% sucrose in PBS at 4° C. Frozen brains were cut at -25°C to obtain 35µm-thick sections containing rostral medulla regions. Free-floating immunohistochemistry (IHC) was performed using anti tyrosine-hydroxylase (TH) primary antibody (TH, Novusbio, NB300-109, 1:5000) and diaminobezidine as a chromofor (ABC Vectastain kit, Vector Laboratories, Peterborough, UK). The resulting images were analyzed in Fiji (ImageJ) using a multi-point counting tool.

For each brain, the sum of immunopositive cells counted in five sections with the same position of the RVLM was calculated and used for statistics.

Telemetry to measure cardiovascular function in conscious, unrestrained rats
Three-month-old male SHR (body weight: 277 ± 6 g, n = 7) and SHR-*Tert*$^{-/-}$ (body weight: 217 ± 10 g, n= 5) were implanted with radiotelemetry devices (model HD-S10, Data Sciences International, USA) under isoflurane anesthesia (5% for induction and 2.5% for maintenance; Forane, AbbVie, USA). Briefly, the abdomen was shaved and sterilized, and a midline abdominal incision was made. A spreader was used to enlarge the incision, and the intestines were pushed aside. The tip of the telemetry probe catheter was inserted retrogradely into the abdominal aorta through a puncture site, which was then sealed with tissue adhesive (Vetbond™, 3M Animal Care Products, USA). Finally, the intestines were repositioned, and lentocilin (Laboratórios Atral, Portugal) was administered to prevent infection. The transmitter body was secured to the abdominal wall before the midline incision was closed with Michel suture clips (Medin, Czech Republic). One animal (SHR-*Tert*$^{-/-}$) died after device implantation. Transmitted data was recorded and analyzed using the Dataquest A.R.T. system (Data Sciences International, USA). After a 10-day recovery period, systolic blood pressure (SBP), diastolic blood pressure (DBP), mean arterial blood pressure (MAP), pulse pressure (PP), heart rate (HR), body temperature, and activity were measured in freely moving rats under LD12:12 conditions for 10 days. The light was then not turned on, and the rats were kept in constant darkness for the next 10 days (5-minute intervals were recorded every 15 minutes, four times per hour). Missing values in the telemetry data are the result of temporary problems with the implanted radio-telemetry devices.
Telemetry Analysis. Parameters obtained from telemetry were analyzed by three different approaches. First, in order to assess the presence or absence of the circadian rhythms in the measured parameters, we used an open-source online platform BioDare2. Rhythmicity was assessed by eJTK method, where the Benjamini-Hochberg adjusted P-value represents the degree of rhythmicity (data sets with pBH < 0.001 are considered as rhythmic, data sets with pBH > 0.001 are considered as nonrhythmic). Period, circadian phase, and amplitude were measured using mFourfit model. For data on LD12:12 and DD, both rhythmicity and period analysis measurements were processed separately, and no data preprocessing was performed. Next, we performed a correlation analysis between all measured parameters. For each animal, correlation matrices were created in Python using Spearman’s correlation coefficient. To further investigate the relationship between spontaneous activity and cardiovascular parameters, we calculated the mean of each parameter during the active or rest phase, and the amplitude as half of the difference between the maximum and minimum during each circadian cycle. Day-night dipping analysis was done by calculating the percentage difference between the subjective night and the following subjective day.

SUPPLEMENTARY TABLES

Supplementary Table S1. Telomere size statistics

Table includes results of various parametric and non-parametric tests in order to compare telomeres length in different tissues in SHR and SHR-*Tert*$^{-/-}$ rats. N=5 for both groups for all tissues. MD – mean difference, CI – confidence interval.

Tissue	MD	CI lower limit 95%	CI upper limit 95%	Permuta-tion test	Welch's t-test		Student's t-test		Mann–Whitney U-test	
				p value	p value	statistic	p value	statistic	p value	statistic
spleen	-0.4052	-0.5221	-0.3195	0.0006***	0.00038***	7.28105	0.00009****	7.28105	0.01219*	25
liver	-0.3856	-0.5513	-0.1543	0.0218*	0.00894**	3.43389	0.0089**	3.43389	0.03671*	23
kidney	-0.2947	-0.3685	-0.1857	0.0082**	0.00064***	5.77848	0.00042***	5.77848	0.01219*	25
heart	-0.2289	-0.435	-0.1011	0.0082**	0.05282	2.41829	0.04196*	2.41829	0.02157*	24
intestine	-0.3296	-0.6	-0.0992	0.0072**	0.07576	2.31383	0.04939*	2.31383	0.02157*	24
skin	0.2256	-0.7108	0.7285	0.585	0.57617	-0.5965	0.56736	-0.5965	0.29627	7

Supplementary Table S2. List of primers for RT qPCR

	primer	sequence
B2m	Forward	CGCTCGGTGACCGTGATCTTTCTG
B2m	Reverse	CTGAGGTGGGTGGAACTGAGACACG
Gapdh	Forward	TGATTCTACCCACGGCAAGTT
Gapdh	Reverse	TGATGGGTTTCCCATTGATGA
Tbp	Forward	CATCATGAGAATAAGAGAG
Tbp	Reverse	GGATTGTTCTTCACTCTTG
Per1	Forward	CGCACTTCGGGAGCTCAAACTTC
Per1	Reverse	GTCCATGGCACAGGGCTCACC
Per2	Forward	GAATTTTCACAACAACCCAC
Per2	Reverse	TGTAGGATCTTCTTGTGGATG
Bmal1 (Arntl)	Forward	ATGAAAACATTGAGAGGTGC
Bmal1 (Arntl)	Reverse	GGATCTTGAAGACAGATTCG
Nr1d1 (Rev-ErbA)	Forward	GCTGTGCGGGAGGTGGTAGAAT
Nr1d1 (Rev-ErbA)	Reverse	TGTAGGTTGTGCGGCTCAGGAA
Cry1	Forward	GTGGTGGCGGAAACTGCTCTC
Cry1	Reverse	ACTCTGTGCGTCCTCTTCCTGA
Dbp	Forward	TTTGCGCCGCTGCTGTGGGAACG
Dbp	Reverse	GGGGGAGGGCGCGGGAGTGC
E4bp4 (Nfil3)	Forward	GCAGGAGCCCGTGGAGTTGGAGAG
E4bp4 (Nfil3)	Reverse	AGGAGGGGAGGGGAGTGGGAGTAGGT
Nampt (Pbcf)	Forward	CTTTGGTTCTGGTGGCGCTTTGCTAC
Nampt (Pbcf)	Reverse	GCCGGCCCTTTTTCGACCTTTTGTT

Supplementary Table S3. Analysis of circadian rhythms in the heart rate and blood pressure profiles of SHR and SHR-*Tert*$^{-/-}$ maintained on LD12:12 and constant darkness (DD). Analysis was performed on the online platform BioDare2. For each animal, table contains results of the rhythmicity test, period (in hours), circadian phase, and amplitude. Rhythmicity of each measured parameter is shown as a Benjamini-Hochberg corrected p-value obtained from rhythmicity test based on the eJTK algorithm. Parameter is considered to be rhythmic if p-value is less than 0.001. In some animals, due to biosensors issues (SHR1, SHR4) and/or animals decease (SHR7), telemetry was not performed through the whole experiment (marked as N/A in the table).

Parameter	SHR_1	SHR_2	SHR_3	SHR_4	SHR_5	SHR_6	SHR_7	SHR-*Tert*$^{-/-}$_1	SHR-*Tert*$^{-/-}$_2	SHR-*Tert*$^{-/-}$_3	SHR-*Tert*$^{-/-}$_4
**Temperature**											
Rhythmicity, LD	$7.54E^{-76}$	$1.94E^{-83}$	$2.73E^{-84}$	N/A	$8.44E^{-91}$	$2.87E^{-89}$	$1.63E^{-68}$	$4.54E^{-107}$	$2.73E^{-84}$	$8.44E^{-91}$	$8.03E^{-76}$
Rhythmicity, DD	N/A	$9.98E^{-87}$	$2.68E^{-86}$	$5.26E^{-91}$	$9.66E^{-95}$	$2.68E^{-86}$	N/A	$2.08E^{-106}$	$1.51E^{-107}$	$3.97E^{-91}$	$4.48E^{-85}$
Period, LD	23.96	24.1	23.98	N/A	23.98	23.96	24.02	24.02	23.96	24	24.02
Period, DD	N/A	24.08	24.12	24.06	24.04	24.1	N/A	24.02	24.06	24.06	24.04
Phase, LD	14.24	12.75	14.05	N/A	13.37	14.02	13.77	13.6	14.05	13.52	13.11
Phase, DD	N/A	22.26	21.5	22.47	21.74	21.3	N/A	22.85	23.19	22.7	21.63
Amplitude, LD	0.51	0.5	0.58	N/A	0.6	0.59	0.7	0.78	0.58	0.76	0.51
Amplitude, DD	N/A	0.54	0.5	0.6	0.56	0.56	N/A	0.6	0.7	0.46	0.55
**Heart rate**											
Rhythmicity, LD	$8.52E^{-49}$	$3.63E^{-53}$	$1.08E^{-59}$	N/A	$4.45E^{-57}$	$2.25E^{-59}$	$2.82E^{-47}$	$1.01E^{-66}$	$3.82E^{-71}$	$1.52E^{-44}$	$1.58E^{-60}$
Rhythmicity, DD	N/A	$1.56E^{-53}$	$3.37E^{-62}$	$7.37E^{-74}$	$7.68E^{-57}$	$5.80E^{-57}$	N/A	$1.83E^{-76}$	$5.51E^{-75}$	$7.55E^{-56}$	$8.28E^{-66}$
Period, LD	24.02	24.08	24.04	N/A	23.98	23.98	24.02	24.08	23.94	24	24.08
Period, DD	N/A	24.12	24.08	24.02	24.1	24.02	N/A	24.02	24.12	24.1	24.08
Phase, LD	13.37	11.83	13.25	N/A	12.47	13.07	13.1	12.55	13.77	12.17	12.04
Phase, DD	N/A	10.95	11.89	11.94	10.8	12.15	N/A	13.36	11.64	9.72	12.21
Amplitude, LD	34.53	35.26	32.41	N/A	31.66	43.89	39.77	39.4	31.66	35.37	37.51
Amplitude, DD	N/A	30.9	29.97	36.71	28.49	37.15	N/A	37.91	25.19	31.39	35.81
**Mean arterial pressure**											
Rhythmicity, LD	$4.03E^{-05}$	$1.71E^{-06}$	$2.40E^{-06}$	N/A	$1.07E^{-06}$	$2.31E^{-08}$	$1.09E^{-06}$	$6.49E^{-10}$	$4.31E^{-12}$	0.00102	0.06552
Rhythmicity, DD	N/A	$4.87E^{-07}$	$5.01E^{-06}$	$4.87E^{-07}$	0.04048	0.00178	N/A	0.00375	$5.38E^{-04}$	$5.38E^{-04}$	0.05801
Period, LD	24.1	24.06	24.18	N/A	24	23.9	24.08	24.04	23.88	24.04	24.02
Period, DD	N/A	24.1	24.08	23.98	24.08	24.08	N/A	24.04	24.12	24.12	24.06
Phase, LD	15.26	13.68	13.16	N/A	12.86	14.44	14.02	12.48	12.17	7.15	8.51
Phase, DD	N/A	12.16	14.02	13.83	12.41	11.39	N/A	12.18	8.16	5.14	10.7
Amplitude, LD	2.62	3.48	3.13	N/A	2.12	3.46	4.5	3.26	3.98	2.14	0.59
Amplitude, DD	N/A	3.14	2.6	3.71	1.43	2.3	N/A	1.67	2.43	2.28	0.99
**Systolic blood pressure**											

Rhythmicity, LD	$5.06E^{-05}$	$1.35E^{-07}$	$3.90E^{-09}$	N/A	$8.15E^{-12}$	$1.20E^{-10}$	$7.44E^{-08}$	$8.15E^{-12}$	$4.19E^{-09}$	0.01032	0.47473
Rhythmicity, DD	N/A	$2.52E^{-09}$	$4.98E^{-09}$	$1.17E^{-12}$	$8.27E^{-05}$	$2.52E^{-05}$	N/A	0.00107	0.04429	0.05699	0.30280
Period, LD	24.1	24.06	24.18	N/A	23.98	23.9	24.06	24.04	23.86	24.04	24.06
Period, DD	N/A	24.14	24.06	24	24.14	24.16	N/A	24.04	24.12	24.12	24.08
Phase, LD	16.02	14.51	13.81	N/A	14.31	15.63	15.17	14.03	13.35	9.97	22.51
Phase, DD	N/A	12.5	14.83	14.19	12.17	11.83	N/A	15	8.75	5.92	18.24
Amplitude, LD	3.56	4.1	4.42	N/A	3.6	4.64	5.21	4	3.69	1.09	0.76
Amplitude, DD	N/A	4.23	4.22	5.1	2.62	3.22	N/A	2.26	1.97	1.59	0.34

Diastolic blood pressure

Rhythmicity, LD	$1.40E^{-06}$	$2.47E^{-07}$	$2.32E^{-05}$	N/A	$1.04E^{-04}$	$2.47E^{-07}$	$5.32E^{-07}$	$5.70E^{-09}$	$1.51E^{-16}$	$1.87E^{-06}$	0.00232
Rhythmicity, DD	N/A	$6.41E^{-06}$	$1.46E^{-04}$	$8.65E^{-08}$	0.15689	$1.46E^{-04}$	N/A	$1.29E^{-04}$	$6.92E^{-08}$	$6.92E^{-08}$	0.00218
Period, LD	24.08	24.08	24.08	N/A	24.04	24.06	24.18	24.14	23.86	24.04	24.06
Period, DD	N/A	24.02	24.08	24	24.12	24.08	N/A	23.92	24.12	24.12	24.06
Phase, LD	14.36	12.21	12.91	N/A	10.6	11.88	12.63	9.82	11.51	6.67	8.29
Phase, DD	N/A	12.26	12.65	12.6	10.47	9.42	N/A	11.32	7.53	4.89	9.84
Amplitude, LD	2.39	3.2	2.36	N/A	1.8	3.15	4.29	2.94	4.27	3.07	1.4
Amplitude, DD	N/A	2.66	1.65	3.38	1.04	2.33	N/A	1.89	2.88	2.97	1.64

Pulse pressure

Rhythmicity, LD	$4.01E^{-07}$	$6.56E^{-23}$	$3.80E^{-31}$	N/A	$5.42E^{-27}$	$1.02E^{-36}$	$5.97E^{-24}$	$3.95E^{-41}$	$1.28E^{-17}$	$8.07E^{-24}$	$1.40E^{-15}$
Rhythmicity, DD	N/A	$5.14E^{-28}$	$2.72E^{-32}$	$1.39E^{-46}$	$1.71E^{-20}$	$1.68E^{-33}$	N/A	$4.77E^{-30}$	$1.40E^{-04}$	$1.15E^{-11}$	$1.61E^{-12}$
Period, LD	24.4	24.04	23.92	N/A	24	23.96	24.02	23.86	23.86	23.96	24.02
Period, DD	N/A	24.1	24.08	23.88	23.88	23.98	N/A	24.24	24.1	24.06	24.14
Phase, LD	17.59	18.04	16.77	N/A	16.16	17.94	18.94	17.97	19.53	17.81	21.31
Phase, DD	N/A	15.59	15.51	18.61	17.79	17.98	N/A	14.14	17.82	16.78	19.84
Amplitude, LD	1.84	2.24	2.5	N/A	2.78	2.98	2.7	3.37	1.99	2.5	2.08
Amplitude, DD	N/A	2.47	2.86	2.44	1.89	2.75	N/A	2.9	1.18	1.51	1.88

Supplementary Table S4. Statistical comparison of gene expression in the peripheral tissues of SHR and SHR-*Tert*$^{-/-}$
Results of 2-way ANOVA with a Holm-Šídák post hoc multiple comparison test

**ATRIUM**	**DD1** CT6 vs. CT18		**DD14** CT6 vs. CT18	
**gene**	SHR	SHR-*Tert*$^{-/-}$	SHR	SHR-*Tert*$^{-/-}$
Per1	P = 0.3315	P = 0.8122	P = 0.0111	P = 0.2521
Per2	P = 0.6552	P = 0.5901	P = 0.5694	P = 0.9105
Cry1	P = 0.9979	P = 0.9016	P = 0.0196	P = 0.4598
Nr1d1	P = 0.0056	P = 0.0189	P < 0.0001	P = 0.1033
Bmal1	P = 0.9113	P = 0.0057	P = 0.0136	P = 0.1823
E4bp4	P = 0.8887	P = 0.7900	P = 0.0059	P = 0.0870
Dbp	P = 0.0844	P = 0.1448	P = 0.0122	P = 0.0721
Nampt	P = 0.4713	P = 0.5223	P = 0.0772	P = 0.9829

**LIVER**	**DD1** CT6 vs. CT18		**DD14** CT6 vs. CT18	
**gene**	SHR	SHR-*Tert*$^{-/-}$	SHR	SHR-*Tert*$^{-/-}$
Per1	P = 0.0045	P = 0.2013	P = 0.7870	P = 0.6216
Per2	P = 0.0187	P = 0.2613	P = 0.0320	P = 0.4877
Cry1	P = 0.0008	P = 0.0200	P = 0.0027	P = 0.0766
Nr1d1	P < 0.00001	P < 0.00001	P = 0.0184	P = 0.3207
Bmal1	P = 0.0788	P = 0.3142	P = 0.0072	P = 0.0521
E4bp4	P > 0.9999	P = 0.8864	P = 0.0049	P = 0.4791
Dbp	P = 0.0552	P = 0.0340	P = 0.2948	P = 0.4817
Nampt	P < 0.0001	P = 0.0080	P = 0.5767	P = 0.9744

**gWAT**	**DD1** CT6 vs. CT18		**DD14** CT6 vs. CT18	
**gene**	SHR	SHR-*Tert*$^{-/-}$	SHR	SHR-*Tert*$^{-/-}$
Per1	P = 0.0526	P = 0.9865	P = 0.3518	P = 0.3209
Per2	P = 0.2490	P = 0.6319	P = 0.8177	P = 0.3287
Cry1	P = 0.0021	P = 0.0254	P = 0.0010	P = 0.0205
Nr1d1	P < 0.00001	P < 0.00001	P = 0.0127	P = 0.1681
Bmal1	P = 0.3706	P = 0.0021	P = 0.0398	P = 0.2257
E4bp4	P = 0.0029	P = 0.3907	P = 0.0045	P = 0.1310
Dbp	P = 0.0305	P = 0.2435	P = 0.0509	P = 0.1643
Nampt	P = 0.2451	P = 0.9655	P = 0.7062	P = 0.1231

**PANCREAS**	**DD1**<br>CT6 vs. CT18		**DD14**<br>CT6 vs. CT18	
**gene**	SHR	SHR-*Tert*$^{-/-}$	SHR	SHR-*Tert*$^{-/-}$
Per1	P = 0.0883	P = 0.5459	P = 0.9762	P = 0.8845
Per2	P = 0.0065	P = 0.0188	P = 0.0924	P = 0.5287
Cry1	P = 0.0772	P = 0.0029	P = 0.0143	P = 0.2522
Nr1d1	P = 0.0054	P = 0.0322	P = 0.0027	P = 0.2300
Bmal1	P = 0.2307	P = 0.7347	P = 0.0902	P = 0.3727
E4bp4	P < 0.00001	P = 0.0006	P = 0.0045	P = 0.0242
Dbp	P = 0.0002	P = 0.0088	P = 0.1274	P = 0.4388
Nampt	P = 0.9357	P = 0.9045	P = 0.9907	P = 0.1540

SUPPLEMENTARY FIGURES

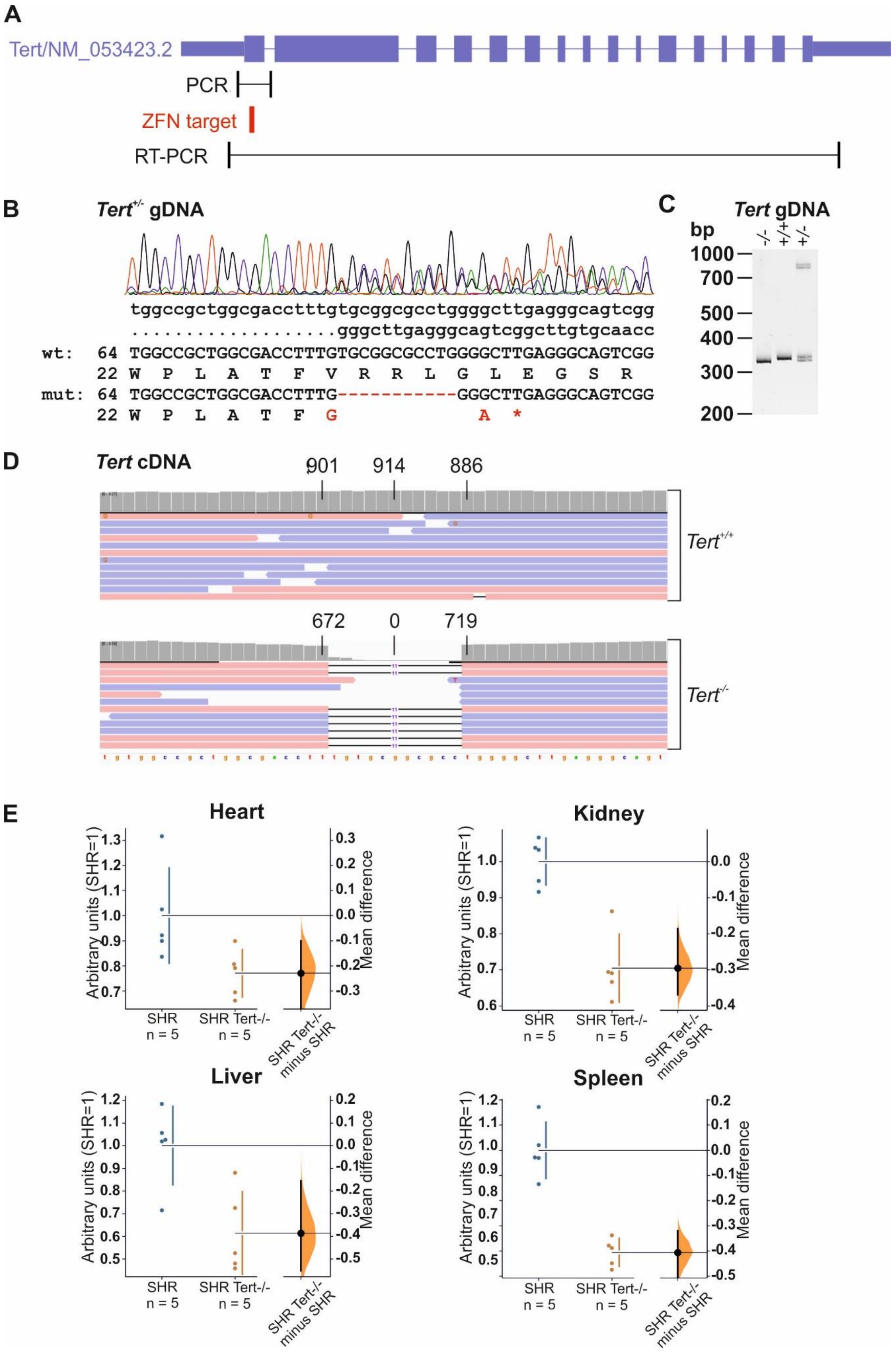

Supplementary Figure S1. Generation of *Tert* deficient rats and telomere size. (A) Schematic depiction of *Tert* gene with relative position of the ZFN target and the PCR used to detect the mutation. Exons (tall boxes) including the UTRs (narrow boxes), PCR and ZFN target are relatively to scale, introns (line) are not to scale. **(B)** Sequencing of the founder #63 revealing a 11 bp deletion. wt = wild type/reference sequence, mut = the mutated sequence. Translation in single-letter code is provided below the nucleotide sequence. **(C)** Genotyping of the deletion using PCR (positioned as depicted in A) and acrylamide electrophoresis. **(D)** Alignment of the cDNA sequencing reads of *Tert*$^{+/+}$ and *Tert*$^{-/-}$ to *Tert* mRNA reference (NM_053423.2) as visualized by Integrative Genomic Viewer. RNA was isolated form liver samples. The columns represent coverage of a representative sample (count highlighted for positions c.80, c.86 and c.92). All cDNA bridging reads contain the 11-bp deletion that was identified in genomic DNA. **(E)** Telomere sizes in SHR and F3 SHR-*Tert*$^{-/-}$. The mean difference between SHR and SHR-*Tert*$^{-/-}$ is shown in the Gardner-Altman estimation plot. Both groups are plotted on the left axes; the mean difference is plotted on a floating axes on the right as a bootstrap sampling distribution. The mean difference is depicted as a dot; the 95% confidence interval is indicated by the ends of the vertical error bar. Detailed statistics for the telomere size comparison is available in the Supplementary Table 1.

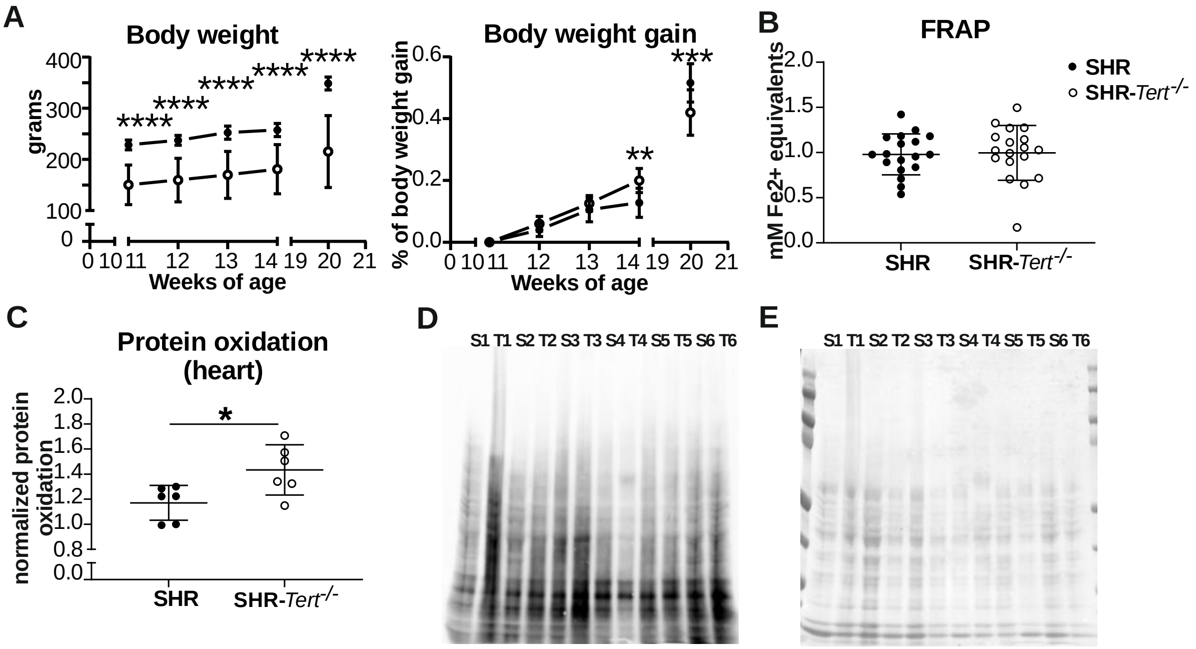

Supplementary Figure S2. Body weights of SHR and SHR-*Tert*$^{-/-}$ rats, Serum Ferric Reducing Antioxidant Power (FRAP), and Normalized level of protein oxidation in the heart apex. (A) Body weights and weight gaining of SHR and SHR-*Tert*$^{-/-}$ rats at different ages (n=8 for each group for 11-14 weeks of age, n=6 for each group for 20 weeks of age) analyzed by 2-way ANOVA. **(B)** Serum Ferric Reducing Antioxidant Power (FRAP) in 5-month-old rats. N=18 for each group, comparison was done using t-test. **(C)** Normalized level of protein oxidation in the heart apex of 5-month-old rats. **(D)** Representative membrane with carbonyl groups in proteins side chains detected by chemiluminescence. **(E)** Representative membrane (the same as in D) stained with Ponceau S. Samples from SHR animals are marked with S, and samples from SHR-*Tert*$^{-/-}$ with T. N=6 for each group, comparison was done using t-test.

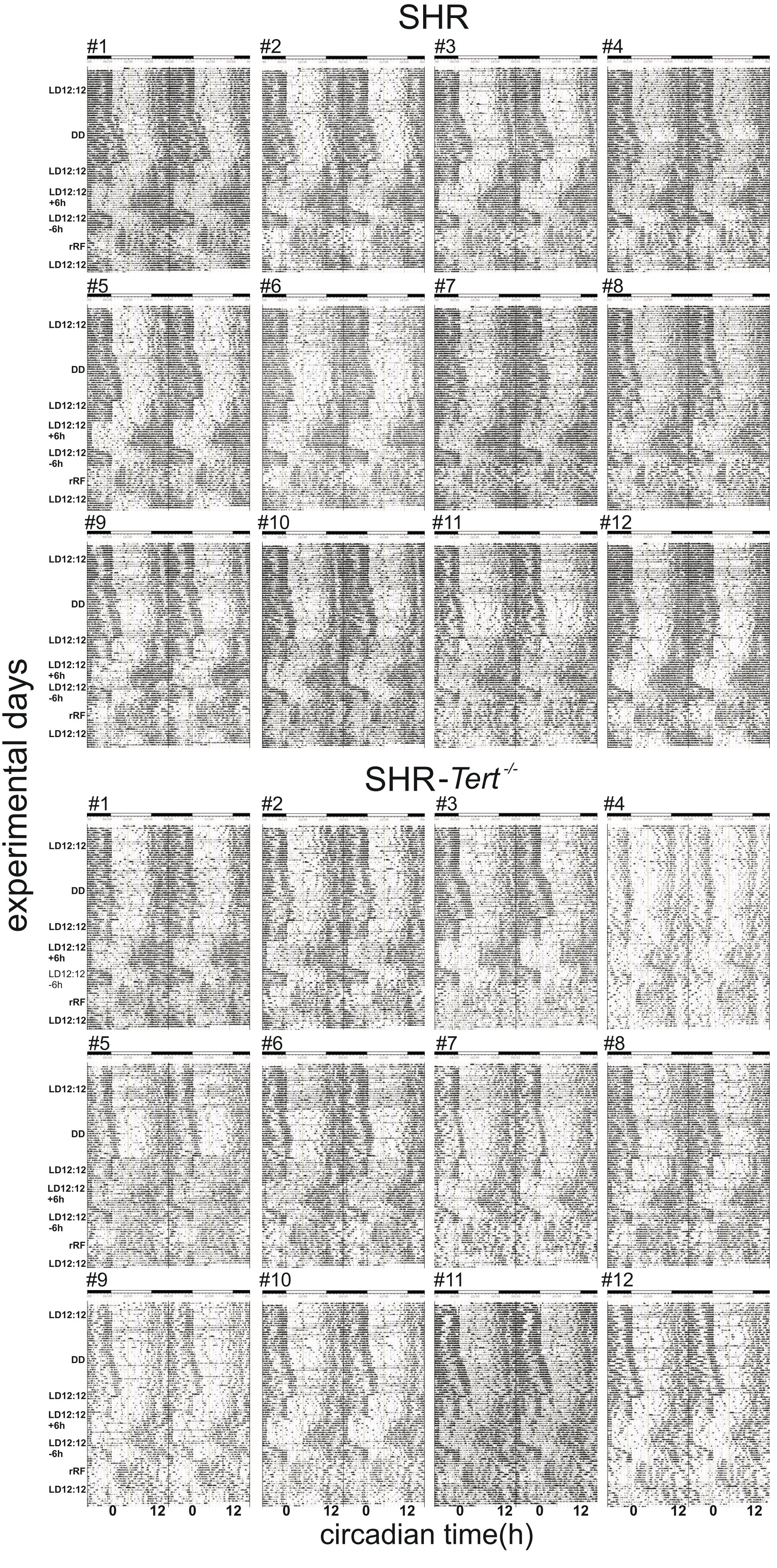

Supplementary Figure S3. Actograms recording. Activity records of individual SHR and SHR-Tert over 90 days of experimental protocol (scheme of the protocol is presented on Fig.1A)

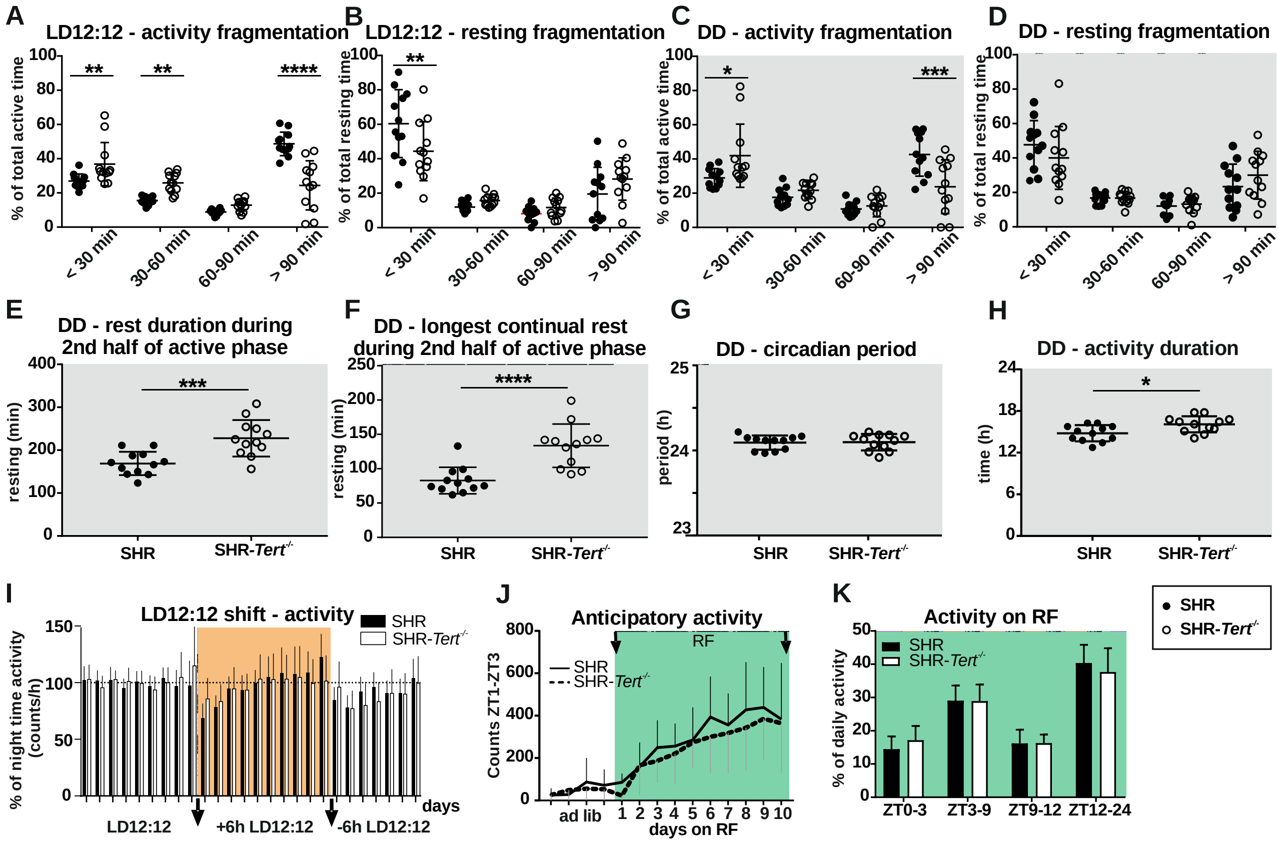

Supplementary Figure S4. Detailed analysis of the locomotor activity. (A) Activity fragmentation on LD12:12. **(B)** Resting fragmentation on LD12:12. **(C)** Activity fragmentation on DD. **(D)** Resting fragmentation on DD. **(E)** Resting time during the second half of the subjective night on DD. **(F)** The longest continual resting interval during the second half of subjective night on DD. **(G)** Circadian period in locomotor activity on DD. **(H)** Duration of the active part of the circadian cycle on DD. **(I)** Synchronization time after a 6-hour phase shifts. **(J)** Anticipatory activity on the reversed restricted feeding (rRF) regimen measured during 2 h before food was provided for 6 h between ZT3-ZT9 on the LD12:12.$^{-/-}$. **(K)** Activity on reversed restricted feeding regime (RF) measured in ZT0-3 (before food), ZT3-9 (during food presence), ZT9-12 (after food removal) and ZT12-24 (during night) intervals. Data are presented as individual values with mean ± SD, t-test (E-H) or 2-way ANOVA, * p<0.05, **p<0.01, ***p<0.001. N=12 animals per group.

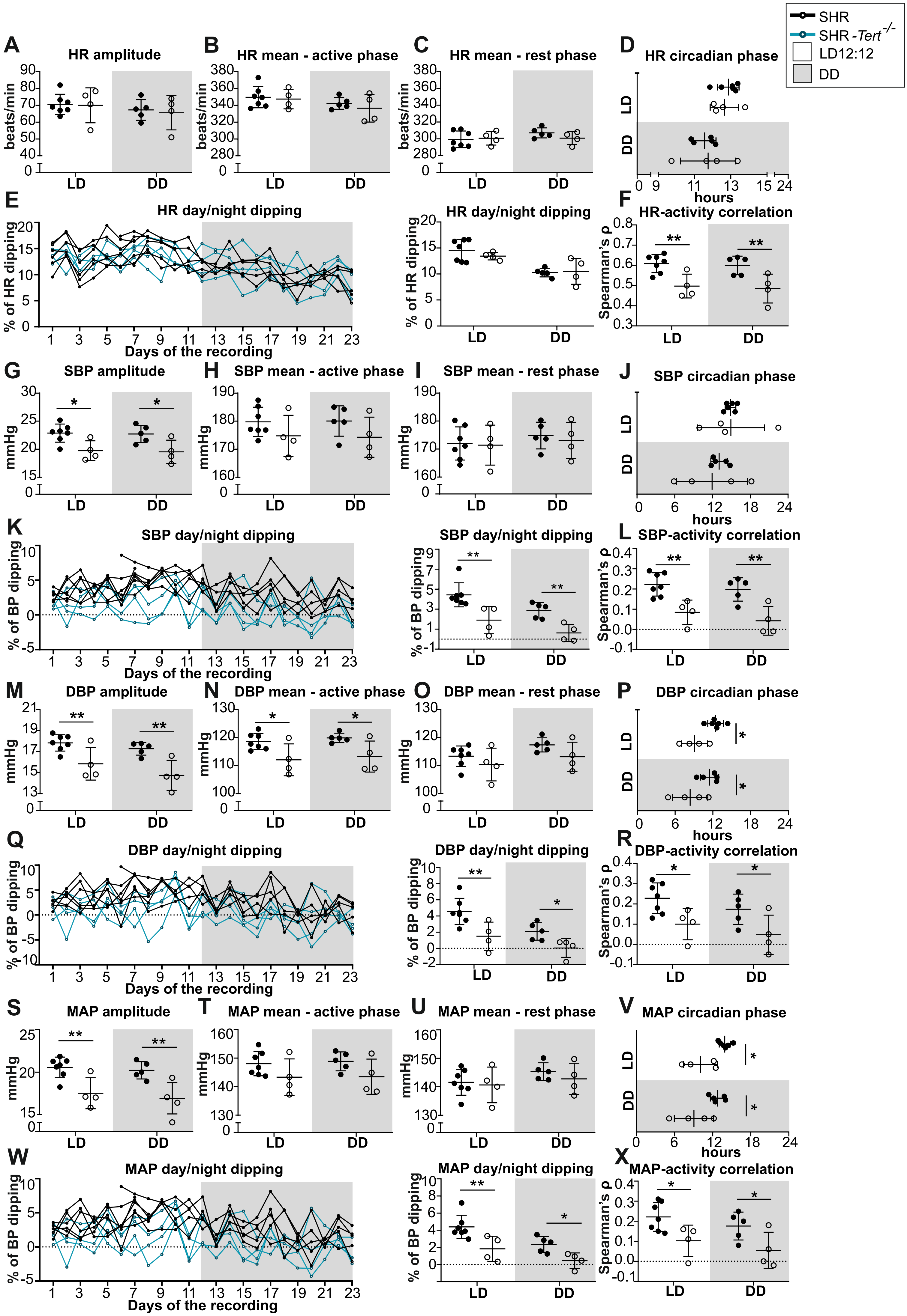
SHR
SHR-Tert-/-
LD12:12
DD
A HR amplitude
B HR mean - active phase
C HR mean - rest phase
D HR circadian phase
E HR day/night dipping
HR day/night dipping
F HR-activity correlation
G SBP amplitude
H SBP mean - active phase
I SBP mean - rest phase
J SBP circadian phase
K SBP day/night dipping
SBP day/night dipping
L SBP-activity correlation
M DBP amplitude
N DBP mean - active phase
O DBP mean - rest phase
P DBP circadian phase
Q DBP day/night dipping
DBP day/night dipping
R DBP-activity correlation
S MAP amplitude
T MAP mean - active phase
U MAP mean - rest phase
V MAP circadian phase
W MAP day/night dipping
MAP day/night dipping
X MAP-activity correlation
beats/min
mmHg
% of HR dipping
% of BP dipping
Spearman's ρ
hours
Days of the recording
LD
DD

Supplementary Figure S5. Detailed analysis of the telemetrically measured cardiovascular parameters.(A) Amplitude of the day/night variation in HR on LD12:12 and DD. **(B)** Mean HR values during the active phase on LD12:12 and DD. **(C)** Mean HR values during the inactive (rest) phase on LD12:12 and DD. **(D)** Phase of the circadian rhythm in HR on LD12:12 and DD. **(E)** Day/night dipping analysis of the HR. **(F)** Correlation between HR and spontaneous activity. **(G)** Amplitude of the day/night variation in SBP on LD12:12 and DD. **(H)** Mean SBP values during the active phase on LD12:12 and DD. **(I)** Mean SBP values during the inactive (rest) phase on LD12:12 and DD. **(J)** Phase of the circadian rhythm in SBP on LD12:12 and DD. **(K)** Day/night dipping analysis of the SBP. **(L)** Correlation between SBP and spontaneous activity. **(M)** Amplitude of the day/night variation in DBP on LD12:12 and DD. **(N)** Mean DBP values during the active phase on LD12:12 and DD. **(O)** Mean DBP values during the inactive (rest) phase on LD12:12 and DD. **(P)** Phase of the circadian rhythm in DBP on LD12:12 and DD. **(Q)** Day/night dipping analysis of the DBP. **(R)** Correlation between DBP and spontaneous activity. **(S)** Amplitude of the day/night variation in MAP on LD12:12 and DD. **(T)** Mean MAP values during the active phase on LD12:12 and DD. **(U)** Mean MAP values during the inactive (rest) phase on LD12:12 and DD. **(V)** Phase of the circadian rhythm in MAP on LD12:12 and DD. **(W)** Day/night dipping analysis of the MAP. **(X)** Correlation between MAP and spontaneous activity. All correlation matrices for individual animals are shown in Supplementary Fig. S6. In total, 7 SHR and 4 SHR-*Tert*$^{-/-}$ 4-month-old animals were recorded in LD12:12 for 12 days (white area) and then in DD for 11 days (shaded area). Data are presented as individual values with mean ± SD, 2-way ANOVA, * p<0.05, **p<0.01, ***p<0.001.

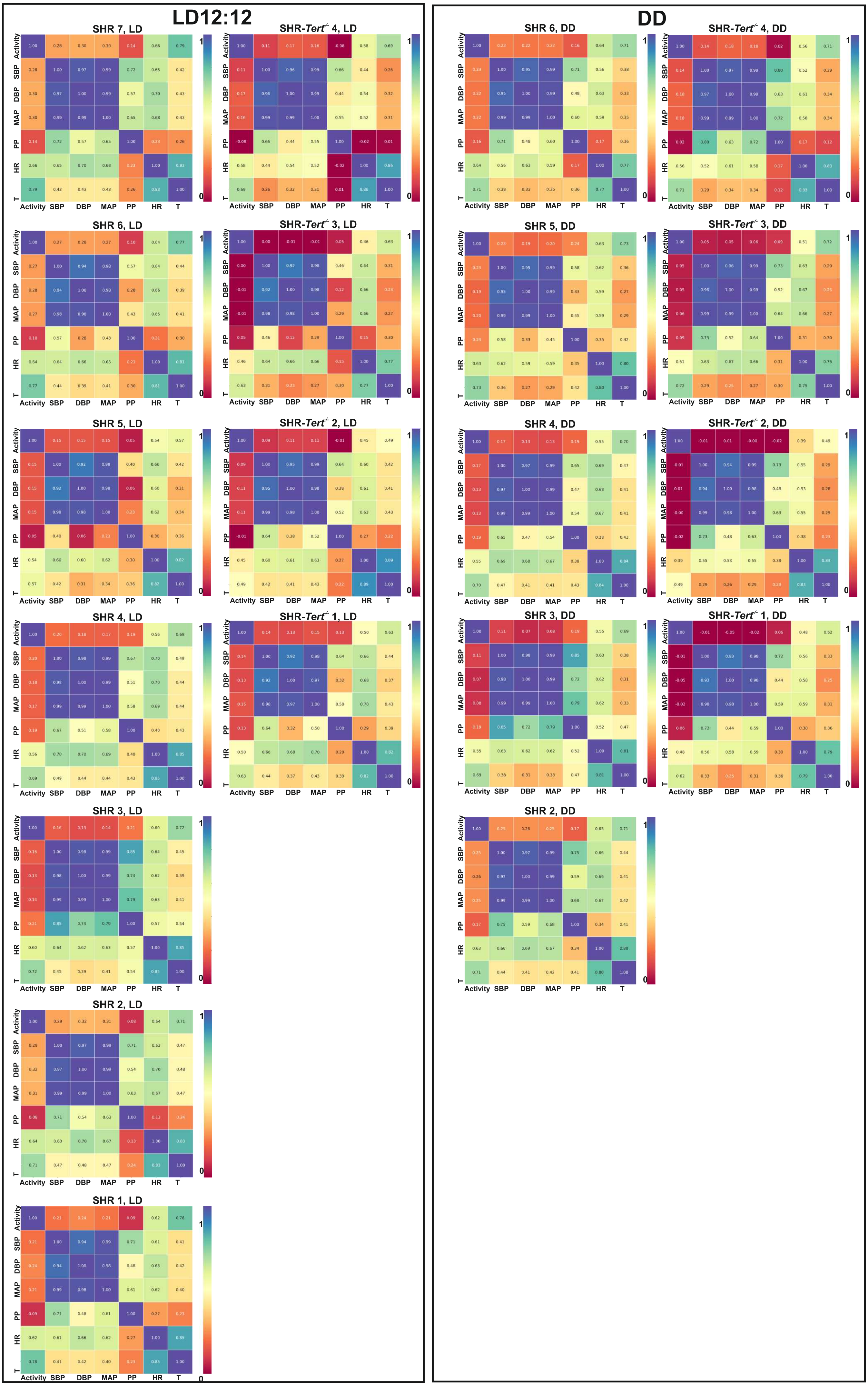
LD12:12
DD
SHR 7, LD
SHR-Tert-/- 4, LD
SHR 6, DD
SHR-Tert-/- 4, DD
SHR 6, LD
SHR-Tert-/- 3, LD
SHR 5, DD
SHR-Tert-/- 3, DD
SHR 5, LD
SHR-Tert-/- 2, LD
SHR 4, DD
SHR-Tert-/- 2, DD
SHR 4, LD
SHR-Tert-/- 1, LD
SHR 3, DD
SHR-Tert-/- 1, DD
SHR 3, LD
SHR 2, DD
SHR 2, LD
SHR 1, LD
Activity SBP DBP MAP PP HR T

Supplementary Figure S6. Correlation matrices for parameters of telemetry of individual SHR and SHR-*Tert*$^{-/-}$.

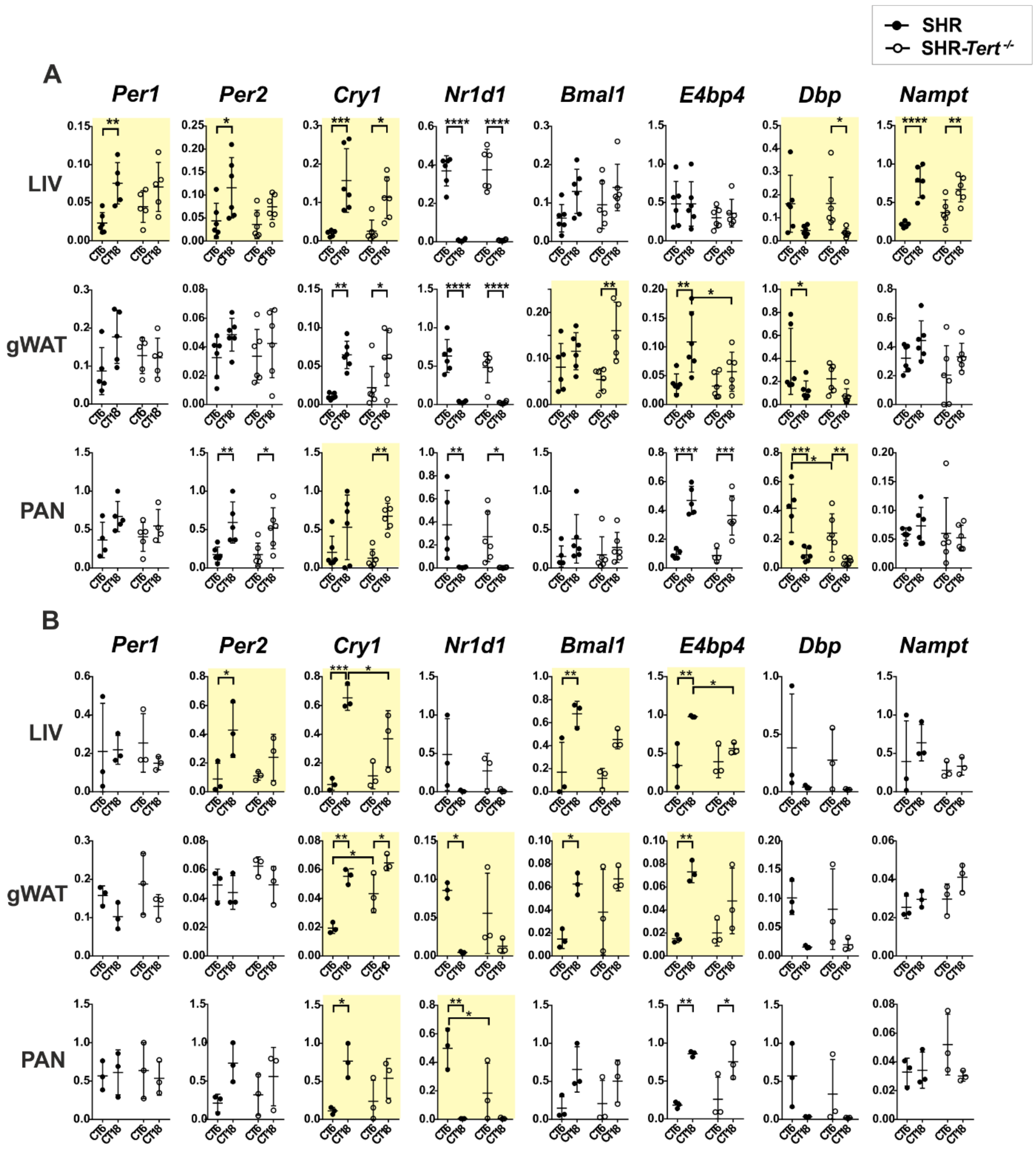

Supplementary Figure S7. Expression of clock genes in the peripheral tissues. Expression of 8 genes (*Per1*, *Per2*, *Cry1*, *Nr1d1*, *Bmal1*, *E4bp4*, *Dbp*, and *Nampt*) was compared in the peripheral tissues collected from 5-month-old SHR and SHR-*Tert*$^{-/-}$ at subjective day (CT6) and subjective night (CT18) **(A)** on day 1 (n = 3-6 per each group and time point) and **(B)** on the 14th day in DD (n = 3 per each group)**.** LIV – liver, gWAT – apididymal fat, PAN – pancreas. For statistics, see Supplementary Table S4. Data are presented as individual values with mean ± SD, 2-way ANOVA, * p<0.05, **p<0.01, ***p<0.001.

SUPPLEMENTARY FILES

Supplementary Data File 1. *Tert* Rat Sequence

Primers bolded and underlined
ZFN binding site in red
ZFN cut site in lowercase red

TTTGGCTGTGTGCAAACTCAAGCGCCCTCCCCTAGAGGCCCTGGCCTCGCTCTGGT
GCCCTAGGGTGAAGGACACTGGAGAGAAGCTAAAGCAAGACTTTCTTCACCTAG
TCCGCTTCCTTTGGAACATATTTGGGATCTGAGTGTGTGATAGTACCACAATAAA
GCCTTAAGAACCTATACAATAGAATCCCGGTTGTAATCATGAAGCCTGGTTGGGA
AAAACTGCGACTGCCACTACCTCACTCACTCACTGTCGATGTCAACCACAGCAGG
CAGCAGCGGCCCGCTCGGGGAGTGGCAAAGCCTTAGGTCCCTCCGCCTACCTAAT
CTTCAATAGGTCAAGGATGGGCTTCTTTGCTTGCCCAGAGAAAAGGGAGCCCGAG
AAGCATTCTGTAGAGAGAAGCCCTGCATGACTGCGCCTCCCTTCGTTATTTCTACA
CTTCCGCCAACCACTGAACTTGGCTGGGAACACACCTGGTCAGTAGTCCCAAGCA
CCAGCTGTGGGACCATCACACAGCTGCGTCCCCGCCCCTTCCGCTACAACGCTTA
ATTCTCTTGGGTCCCGCCCCTTCCTCCGTTCCCGGCCTCCTCTTTTTCCGCCGCGGG
CTCTCAATGGCTGGGATTCTGACGA**TTTTCTAAGCACACCCCTGC**GCCTTGGTTC
CCGCACGTGGGAGGTTCATCCCGACCTAGAGCACGATGCCCCGCGCTCCTCGTTG
CCCCGCCGTGCGCTCTCTACTGCGCAGCCGATATCGGGAGGTGTGGCCGCTGGCG
ACCTTTGtgcggcGCCTGGGGCTTGAGGGCAGTCGGCTTGTGCAACCCGGGGACCCG
AAGGTCTTCCGCACGTTGGTTGCCCAGTGCCTAGTGTGCGTGCCCTGGGGCTCAC
AGCCGCCACCTGCTGACCTTTCCTTCCACCAGGTGGGCCTCCAGGTGGGATCCCC
ATGGGTCGGGGACGGAAAGCGGGAAGGATGCGGGACAG**CGCCTAGCTCACATG**
TCAAGACCCTCTTATCTTTTACCAGGTGTCATCCCTGAAAGAGCTGGTGTCCAGG
GTTGTGCAGAAACTTTGCGAGCGCGGTGAGAGGAATGTGCTGGCTTTTGGCTTTG
CACTGCTTAACGGGGCCAGAGGTGGGCCTCCCATGGCCTTCACGACCAGCGTGCA
TAGCTACTTGCCCAACTCGGTTACTGAGTCCCTGTGTGTCAGTGGTGCATGGATGC
TACTGTTGAGCCGAGTGGGCGACGACCTGCTGGTCTACCTGCTGTCGCACTGTGC
GCTCTACCTGCTGGTGCCCCCCAGCTGTGCCTACCAGGTGTGCGGGTCACCCCTGT
ACCAAATTTGTGCCACCACGGATACCTGGTCCTCTGTGCCCGCTGGTTACAGGCC
CACTCGACCCGTGGGCGGGAATTTCACTAACCTTGGGTCCGCACACCAGATCAAA
AACAGTGGTCACCAGGAAGCACCAAAACCCCAGGCCTTGCCATCACGAGGTACG
AAGAGGCTTCTGAGTCTCACCAGTACAAACGTGCCTTCAGCTAAGAAGGCCAGGT
TTGAACCTGCCCTGAGAGTGGATAAGGGACCCCACAGGCAGGTGGTACCAACCC
CATCAGGCAAAACATGGGCGCCAAGTCCTGCTGCGTCCCCCAAGGTGCCTCCTGC
AGCGAAAAACTTGTCTTTGAAAGGAAAGGCATCTGACCCGAGTCTCTCTGGGTCG
GTGTGCTGTAAACACAAGCCCAGCTCCTCGTCCCTGCTGTCATCACCACCCCAAG
ATGCTGAAAAGCTCAGGCCATTCACTGAGACCAGACATTTCCTTTACTCCAGGGG
AGGTGGCCAAGAGGAGCTAAATCCCTCATTCCTACTCAACAGCCTCCCGCCTAGC
TTGACCGGGGCCAGGAGACTGGTGGAGATCATCTTTCTGGGCTCAAGGCCTAGGA
CATCAGGACCATTCTGCAGGACCCGCCGCCTGCCCCGTCGATACTGGCAGATGCG
ACCCCTATTCCAGCAGCTGCTCATGAACCACGCAAAGTGCCAATATGTCAGATTC
CTCCGGTCGCACTGCAGATTTCGAACAGCAAACCAGCGGGTGCCGGATGCCATGG
ACACCAGCCCATCCCACCTCACGAGTTTGCTCCGGTTACACAGCAGCCCCTGGCA
GGTATACGGCTTTCTTCGGGCCTGCCTCCGCGAGCTGGTGCCTGCCGGTCTCTGGG
GCACCAGGCACAATGAGCGCCGCTTCTTAAAGAACGTGAAGAAGTTCATCTCGTT
GGGGAAGTACGCCAAGCTATCCCTGCAGGAACTGATGTGGAGGGTGAAAGTGGA

GGACTGCCACTGGCTCCGCAGCAGCCCAGGTGAGTGTGGCTGGTGCCCAGCTGAA
TGCACGAGGGGCCCCGGGAGGGGAGACAGTGGGTGACAGTAACCCAGATCCCCG
GTGCTCTGGCAGCTTTATGCAGTCAGTGGTTGGAAGACTTCCATCTGATGGTCTCT
GATACCAAGCTCCCTCCAGCTCACCTTGCACAAACTAAGACTCTTGTCCAAGCCC
TGGGCAGGTTCTCAGTGCTGGGGACATTGTGGTGAACAGTTATGCCGATGGGGAG
CACGGTGGATAGGAGTTCTGGCACCTGGTTAGAGAGAGAGTGAAGTCTCCCAGT
GTGCATTCACTGAAGAATGAGAGTCTGCAGAAGCAGGGAGCAGTTTATTCTGCAG
AAGTCGAGCATGAGGGGGCAGCGAGGGAGGGTGTCTCTCATTCCAAGATGGAGA
GACAAACAAGTGGTCTCGCAGGCTAGATTTAAAGGGGATTCTGGGATAGGTGAC
CTTTATCTTACCCCATCTCTAGGGGCATACCATTACTGAAGCATGTCAGCTGGAA
ACTGTTAGTGGGGAGGTCTGGAAACTTGCTGCCCCATTGTCCCTGCTTCAGGCCA
GGTAGCCAGGCAGCTTCTAAGGGCAGGAGAGTTTCTGACTGGCTACCTAAAAGTT
GTTTGTTTGTTTCTTTGTTTTTCCCTAGTAACGTACTTGTCTGGACTTGCTCAGTTC
TTAGGCCTAGTTTCCTAGACTGCCAATTTGAAGCCTGTAAGGAGTCAGCCTGTCTC
ACTACTCCAGGTTAACCATAATCCCCCTGTGGAATGGTGCCTCACTGATAACAGT
GGGAGTCCAACACAGGAACCCGTTGTCCTTGTGGGAAGTGGGGCGCCTGGGTTTC
AAAGGTTGTTCTGGTTCTCCCAATGTGCAGATGTGTGCTTAAGCTATGATGTCAGT
CCATCCACGGTGTGTAGGGTGAACTAAGGAGAGATAAAAAGACTGTTTCCTACA
GTGGAGATCCCTGTCCCATCTTAGCCATGAGTAGAGACTGGAGAGTAGAGCCTGG
TGTGTAAAAGGGATCTTTGTGTTTCTTGGGGGGCTGCAGAGCCTGGACTCCAGGT
CCCTCCTTGCCTGCTTTTCTGGGTTTAATGTTGAGATTGGCCTCCTGTAGTACTACT
TGGCCCCTTCCCTTTCAGCCGGCCCTTCTGTTACAACCTGTGCCTGTATGGAAGGG
ACCAAATTCTCTATCCTGCTCCCCTTCTCCCAGACCCCTAGGATAGTAACAACCAA
TGGGGAAAAGATGGTAGAGCCCTGTTTACACACCGTGGATGATCTTAGGAAGCTT
CAAAGTAAGACACCTTTAATCCCAGCACTCAGAGGCAGAGGCAGAGGCAGAGGC
AGGTGGATCTCTGAGTTCAAGGACAGCCTGACTTACAAAGCTGGGGGCTCTGATA
GCTACACAGAGAAACCCTATCTCAAAAACAAACAAACCAAAAACAGACCTACCC
TGGAGGTGATAGGGCACACCTTTAATCCTAGCACTCTGGTGGCAGAGGCAGGCA
GGTCTCTAAAGAGAGTTTGGGGCTAGCCTAGTCTACAGGGTGAATTCCAGGACAG
CCTGGGCTACACTCAGAAACCTTATCTTGAAAGAAAACACACCTGACTCTCCATC
TGCAGCACAGACTGTGTGGCTTTTGCAATTCTTCCCACCTGCTGAGCACTGAAGTC
CCCTCCCTTCCAGCTTCTCTGGCTCCCTTACCCACCGCTGTCTGTATCGGTTGCCC
AGGGTTACTGACCCCACCCCCTCACCCCGCCCACCCAAAGAAAGGAGATACTGCA
CTCTGGTTCCTTCCTGCCTCGCCTCGACTAGACCTCGCCAGATCCTACCAGTTTAC
ATCCAGTCTTGTTGTTGCCTTTCTAGACTTGTTTGAACGTCTCTTTCCTGCTCCAAA
GAACCCCCTTTGACCTTCAGTTTGCCTGTGGAGACTTAAGCGTGCTGTTAGAATTT
TATACCTGTCTTCAGGTTTATTCTGGTTCCCCACCACTTTCACTGCCACCCCGACA
TCATAGGTTGGGTTTTTGGCTACTCGTTTCCTCTTGGATACTGCCTTAGCTGTACCT
AGTACTTCTGTATGTGGCCATTTTGATAACTGTGGCTTTTTGGTTTTTGTGTATATG
TGTTTGCGTGTGTGTATTATGTAGAATTTTTCAATCCCAAATGCCCAATATTTTGA
ATTTTCTTTTGAAATTGATTTTGAACTCTGTAATACCAGTAATCGAAGGTGCTGGA
AACTTACACCTCTGCACAGGCAGTTTTCCTAAATGTTCGCTATACTTCTGAGAATT
TTGTGTCCACTATGTCAAATATATACAGGAAAAGTCTGAATCCCCTCTAGTCATG
AAATAGGGCGAGGGCTGCCTTGGTTTCTCGCTGGACAGCTGGGCTTAGTCTTACT
CTGGCAGTTTTACATACAACAGTGTTAGGTGTGTACGAGTTACAAATTTTTATTTA
TCTTATGCTTATTTGAGACAAGGTCTTACTAGGCGGTCCTAGCTGGCCCGTGACTT
GTTTGTAGTTGAGGCTGACCTTGAACTCTCTGAGATCTGCCTGCCCTTGCCTCCCA
AGTGCTGGGATTAAAGATGTGAGCCAGGGCACCCAGCTGGAATTTTTGTCTTTCT
GATTAGAGATTCTTTGATTACTAGAATATAAATTATGTCTCTAGTAATTTTTTTTT
AATGTAAAAGAGGGTTTGGGGTTTTTGTGGGGGTTTTGTTTTTGGGGTTTTTTTGT

TGTTGTTGTTGTTGTTGTTGTTTTGGTTTGGTGTTTTTATTTTGTATTGTTTTGTTTT
GTTTTGAGACAGGTTCTCGATAAATTACTCAAGCTGCCCTCAAATTTACAAGCCC
CTTGCCTCTCAGCTTCTAGGGAAGCTGGGATTAAAGGTGTGCACACCTGAGTTTC
CCATCTCCTCAAACTGACTGCACCTGGCTTGCTTTGCCATCTTCCTGCTATGTCTTC
CCCCCCCCCCCTTTTAAGTTTTGCTTTTAAGATTTTCGAAGCACTGACTGCTCTTCC
AAAGGTCCTGAGTTCAAATCCCAGCAACCACATGGTGGCTCACAACCATCTGTAA
TGGAATCCGATGCCCTCTTCTGGTGTGTCTGAAGACAGCTACAGTGTACTCACAT
ATATAAAAAAATAAATAAATCTTTTAAAAAAAAGATTTTCGGGGGGTTGGGGATT
TAGCTCAGTGGTAGAGCGCTTGCCTAGCAAGCGCAAGGCCCTGGGTTTGGTCCCC
AGCTCCCCCCCCCCCAAAAAAAAAAAAAACAAAAAAAAAATTTCGAATTCTTAG
AATTTAGATGTGTCTTTTCACAGCGTGCAGGTGAATTTTTTATATATAGTCCTGCC
AATTTGACAGTTGCCACTTAGTTGGAAAGCTTAGCTTGTTTGCTTTCTTTCTCTTTA
AGAATGTCTTTAAGCTGTTTCTGTTATTTCCGGTTTGCTTTCATTAGTGTCTTGCGT
GCTTTGCTTGTGTGTATATCTCTGTGCACCGCCTCCATTGCCTGGTAGCTGCAGAA
GCCAGAAGAGAGTATTGAATCTCCCTGAACTGACCTTGTAGATGCCTGACAGCCT
CCATGTGGATGCTGGGATTTAAAACCCAGTCCTTTGAAAAAAAAGTAGCCAGTAT
CTTGACAGCTGGGCCATTTCTTCAGCTGGTTTGCTCTTAACTCTCGCTGTGCCTCT
GTTGTCTTGCTCTCTGAGTAACTGTAAAGTGCATTTCCTTGCTGCAGGTGTTTCCA
TTCTGTCTTTACCTTCTAGCTGTAAGAACTTACGTGCACCATTCTCTGTCAGCAGG
TCAACTGACATTTGAGCCTTAAGGTACTTGGAACTCCGAGTAGCTACTCCATTAG
CTCCTATCCCTCTGCTGATTTAGGTCTGCCTTCAGCCTCTCTGTTCCCAAATCAAA
CATCATTCTTGTTTTCTTAAGGTCACTGCTTGTTTAGTTTTAACCTTCCACGCCATT
TTCTCCATATATTGACCTATTTTTAATGATCATTTTATGTGTAGGAATGTTTTGCCT
GAATGTATGTATGGCGTGCCTGGTGCTGGGTCTGACCACACTGGAACTAGAGCTA
CAGGAAGTTGTGAGCTACCCCAGGGACCTCTGCAACAGCAGCCAGTGCTCTTAAC
TGCTGAGCCAGCTCTCCAGCCCTGTTTCCTTCCTTGTTTGTTCATTCTGTCTTCTAG
CTTGTACCTCCTTCTGGGATTCCCCTTCTCCTTCACAAGTTTCCTTCATATTCATCA
CTGCTGGTAAACCCTCCTTTCCCTGGATAAGGAGGCCCCTGCGGTATCTGGAGAC
ACACAGAGTGGAGCTCACACATCCAGCATGGCAGTAAGAGTTAACCTGTTTTCTA
CTTCATTCTGGACCTCTCTGAACTGTGTTCACTGCTCAGTGGCAATGGGTATGTGT
AGTTGGTGACTTGGTAGTTTAGCAGTAGCTCCCACTGAGGTTTGCCTGTATATGTG
TACGAGGTCTGAGGTTGACGCCGAGTGTCTTCTCTAATCGCCTGGAGCTGATCCT
GCTGGAGTCCGTTTGCCTTTGCCTCCGTAGTGGTACTTGCAGGCACATGTCAGCAC
CCCTGGCTTTTAAAACATGGGTTCTGACGATTAAACTGGGTTTTCGCGCCCCTCCA
GCAAACACTTTGCCAACTGAGCCATGCCCCATCCCCTATTTGAAGACATAATCTG
ATCTAGAGCTGGTAGAAGCTAAGGTTTGCTAAGTCAATCTAGCTCAAAAGAATCA
GTCGATAGTTACAATGGTCCTAGATCGGATTCAGAGATTGATAGTGAATGGCTGT
TGGGGGCTGAAGAATACCTCGAGAGCATTTCTCTGTCAGTCAGGGGTATTTCATC
TGTAGTCAGTAGGCCTGCAGGATTCACCCACTACCGACACGGCCTTGTCAGGAAC
TTTAGTTTACAACATAAAGAAACCAAGCATGCATGCACGCATGCACACACATGCA
CACACACACATTAAATGAGTTTATTAATACATATTATTAGAGCCCAGCATGTCCA
GTCCAGCACCCCATGGGCTCATTTGGGAACCGTGGCTCCTATGCCGGACATTATG
GGTCTGAGGTTGAGTCACAGATGTTGAATATCTGCTACTTTCAGAGAAGGACACT
GTCCCTGCCGCAGAGCACCGTCTGAGGGAGAGGATCCTTGCCATGTTCCTGTTCT
GGCTAATGGACACATATGTGGTACAGCTGCTGAGGTCATTCTTCTACATCACAGA
GACCACGTTCCAGAAGAACCGCCTTTTCTTCTACCGTAAGAGTGTGTGGAGCAAG
CTGCAGAGCATTGGAATCAGGTACCACACAGCCTCTCAGGTATAAGCGTCTCCTA
TTTACATATCCAGAGGCACCGACACCCACACTGTGGCACCTGCCAGCCTGGGCAT
GGACCTTGCAGGCGTGGCCTCCCTGGAGCCATGGACTGGACACCCTGCTTTCTTG
CCTGTGAAAGCATAAGTTGGATCCCTAAAGTAGGAGAGCTTTTTATCAATGGACT

CCACCTTGTGTCTACCCCTGAAGGAAGTGTCTCCTCGGTGGGAGACCACAGGGGT
CCACCCTGAGAAGTGGCAGGGCTCTGAGTGGCTGAGGTTCTGTGCTGATTGAGCT
AGAGTTTGCTGTCTCAGCAGAAGTTTACAAAGGCCAAGCTGAGAGGGCAGAGGA
GCGGCCAGGCAAGGCCCACAGAGAGTGGATTACCATGTTAACTTTGTTTGTTGTT
TTGTTCTTGTTGTCCTGAAACTAGTTCCATAGCCTGGGTTGGCTTCAAACCCCTAA
AGATCCACCTGCCTCTGCCTTCCAAGTGCTGGGATTAAGGGTGTGCTCCCACTAC
CACACAGCTCCGTGTTAGCCTTCTTGAGGTCCAGCCTTAACTATGTATCTCACATC
AGCCATACATTATGCTTGTGGGGATTCTTAACAACCAGCACAGGGCTTCCTGGGG
GCAGGCATGGACACGTTTTGTTTTCATACCTGTGTCTAAGCCAGAATATCCATAA
GAGTCAGCAGATTGCTTCAAAAGTGTCCTGGCCAGTGGGACAACCCCACCCCCAC
CCCCCAAAAGAAGGGAACACCAAGTGGAGACTATGGGACCAGATGTCCCAGGTA
CCAACTCAGGCTAGGTGTCACATGCCCCTCCCAGACAGTTCTATGGGGTGTCTGG
TTGGTGTGGGTTGCTATGGTCCTAGATGTAGCCCCATAGCATGGTTGGGTGTCAA
GCGCTCCAAGTTCCCAAGCGCCCCGATGGGACGTGTCTCGTGAACTATGCATGTC
ATAACTCCACGGTACTCTCTGCAACAGGCAACAGCTTGAGAGAGTTCAGCTACGG
GAACTGTCACAAGAGGAGGTCAAGCATCACCAGGACACTTGGCTGGCCATGCCT
ATCTGCAGATTGCGCTTCATCCCCAAGCTCAATGGTCTCCGGCCCATTGTGAACAT
GAGTTATGGCATGGACACCAGAGCTTTTGGCAAAAAGAAGCAGGTGACTGAACT
TGGCCCCCCCGTACGGCTGGTCGAGTCTATGCTCTGACCTATGTAAGGGCTCATG
AAGTTCTGAGGGAGGCTAAGTAACTTGTTCCTGAGTCTAAAACCTTAGCATGTGG
AAAGGTCTTAAGAGATAGGAGATGGTACCAAGAGACAGAGAGCTTGGGACACAA
GCTCTCAGGGATCTTGCCAGGTAGGGTAGGCACTATATGGCCTCTATGCTGGGAG
GCAAAGTAACCAGGGGCAGCTGCCACCCATCCCCATATCTGGATGTCAGAAGCA
GCTCTGAACCCCACCAGTGCCTGTGTGGTCATTAGTAAGGCCTCAGAGGACAACA
TGAAGCTACTGGTGCCTCAGCTCAGCTGGAAGCCCTTGTAAGGCGACCCTGACCC
TGACTCTAACCAGTGGGCGTGTTTCTAGAAGGCCCGTGGGGGGTGAGTGAGAAC
AGGTTGAGGCAGGTTGGATAAGGGTCCCAAATACAGTCAGTTGACTTAGTGAGGT
AGGTCTTCTAAGACTCTGGCTCTGAGCTGAGGTTCTGGGCTCAGAAATCAGGGTG
GTCAGGGAATGGTCTGCCGGGTCCCTTTCTGATGTGTTTTCTGTGGCCCTGGCTCA
GACCCAGTGTTTCACTCAGAGTCTCAAGACTTTGTTCAGCGTGCTCAACTACGAG
CGGACCAAACATCCTAACCTTATGGGTGCTTCAGTACTGGGTACGAGTGACAGCT
ACAGGATCTGGCGGACCTTCGTGCTGCGTGTGCGTGCTCTGGACCAGACACCCAG
GATGTACTTTGTTAAGGTCATCATTTGGACTCCTGTCTACCACATTTGCTCTCATG
GTCTGGGTTCTAGGGACCTCCTTGGAATAGCCAACACTGGGTGACCTGTTGTACC
TCAAGCAGGTGATTGGGAGTGCTGGCCTGTGAGTGCTTACAGGCTAAAAGTGTTA
GGGCCTCCAGTTCTCTCAGGATCGGGGACCCCACTTTTTGGGGGGCGGATAACTA
GCTCTCAGAACAGTCTTGAGCTCTATATGGGTTCCCTGGACAGTACATTCCAACT
GCTCTGGGTTCTGCTCTGTGGGAGATCCCTCTGGAGGCTGGGTCCTATTCAGAGA
AGTAGCCAAGCCTGACTTACCACCAGGGCCTGGACGCTTCAGTATCTGGGTTAGC
CAGGGGCCCTGTAGGCCCCAAGGTTCTTCAAGGATAATGTAGGCTTTCCTTGTCC
TACAGTCCTTAGTCTGGGGCAATGGGATAGAGAGGGGACTATAGGCCCACTATGC
CCCCAGGGTCCTTTGTCTGGAACAGGAAATTTGTGAGATGGGAATGTGAGTGGCG
TAGTAGAGAAGCATGGAGTTTCCTCCTGCATCTCTCTGTCTCCTGCAGTTTGCTCT
GGGCAGGTTGGAACCCAAGCACCAAGGACAGAGCCAGCAAGGACCCCTGAACCT
GGACCTAACCATGCTTAGTAATACTCGCATGTATTCCACAGGCAGATGTGACAGG
GGCCTATGATGCCATCCCCCAGGACAAGCTCGTGGAAATTGTCGCCAATATAATC
AGGCGCTCAGAGAGCATGTACTGTATCCGCCAGTATGCAGTGGTTCAGAAAGATA
GCCAAGGCCAAGTCCACAAGTCCTTCAGGAGACAGGTAAGACTATAGCGGACTC
TTGTCAGTACTGTGAGCATTTTAGTGGCACAGGCCTGTTCACGTCATGGATATCTA
GCCTCTGTCAGTGACAGAGGTATGGCTGTGATAGGTCCATGTTGTGGCATGTCCA

TGGCGGGGCAGCACATGGTGCCAGTGATCATTTGTCTGACCCAGCCTGCCTCAAT
CTGCGGGAACTCTGGACGGAGCGTCCCCTTTCAGGAGATACCTCCAAATCTCTGG
GGCAGCTAAATGATTTATGCATCCAACGACCTCCAGGGGGAAGGCGGGGTCTGC
AGGTTTAAAGGAGGAAGAGTTTGCACTTAGGCCTGGGAGAGGCACAATGCCTCT
CCCTGAAGTAGGCTATACAAGGCCCACAGGCAAGCCGTATACATGTGTGACAGT
GGCCATGTGCCTTCTGCGCCAAGGGCATAGCCACAGTTACTACAGGGGATTCCTT
CCCTACTTCCAACCAGAGCAAGCACCCTGGGCCTGTGGCGAGGTTAGAGGACAA
CATGCAGGAGTTAGCTTCCTCCACCTACATGCGGGTTCTGGGAATTGAACTAAGT
CATTAGGTTTGACCCTGGGTGTCTTTACTTGTGGAGCCTTTGAGGCACCAGCCCCA
CGTAAACACCTGAAGTTGCACATTTCTCTCTCTAATGGCCCTCAGTCGTGTTGCAC
CCATTCTGGCATGTTGCCAGCTCATTATCATCCTGTTCAAAATCTTCATTTATTTAT
CATAATTTTTTCAGATAACCTCTTGGTTATTTGAAAGCGTGTTACTTAGTTATTTTG
AAAGTGCAGGCATTTTCTATATGTCTTGCTTCTCTTTTCTAGGTTAGTTCTTGTGCT
TAAACTACTCTGGGTGACTTTACTCCTGACACTACAACGAACCCACTTGGCACAT
GGGGTTTTTGTTTATTTTGTGCTATCACATGCTAATTGTGCACAGATTTCATTACG
ATAGCTTCATGAGTGTGTATAATGCGTTTGACCGTATTCACACTTGCAGCCTAACG
TGGTAAATTTTAAAGATCAAGGTCAGTGTGTCATCTCAAGCCATGAGAGGATGGA
GCAGCAGGATTGTAAGCTTGAGGACAGCGGGGCTACACAGAGCCTGCAGTGTTC
ACGCCCCTAGATGCCCGTACCTGGCACGCCCGGGCTTGGTCAGGTGTCTTACCAG
CAGATGGTGCTGTATTCCTTCTGCTGACATAATAAAAGTCAGGCCTCCTAGAGAG
TCTGCTACAGAAGTTGAAGGAGCCATCCCCACTCCCTACCCCTGTCAACTCTTCCT
CCTGGCCACACATTGTCTCTGTGGCGAAGTCAGGGTGCTGCTTGTGTTGCCATCAT
TGGTAACCACAGTTATGGAGATGCCTGGCCTGAGAGAAGCTGACCGTGGTGAAG
GCTGCTGTTTCTGAGGCGCCTCCCGCTGGGGTGCTCGTTCCCACACAAGCCTTTGC
AATGGAGTGGTCCCACGTAGTCCTTTTGTTGGGTTGGACTGTGCCTGTCCTGGCTA
GAAAACCATGTGCCCCTGTTTCCCTCAAGTCTCCACCTCTTCTTTGCTACAGGTCT
CCACCCTCTCTGACCTCCAGCCATACATGGGCCAGTTCACCAAGCATCTGCAGGA
CTCAGATGCCAGTGCACTGAGGAACTCTGTTGTCATCGAGCAGGTCCTCAGACTT
CCTATTAACAAGCCACCCCTGGGGGGAACCTTCCAGCTTTACTTGGCTGTTTGCTT
CAGTGCAGGGATGTGGCTGATGTGCTTCTTGGTAGTGGGTCCCATGAACAAGGGT
AGCAGGACTGGTAGCAGTCCTCATGACATAACCAGAACCCTCCAACTTAGGTGGC
CATGCTAAGAGTTGCTTTGAGGTGCCCTTCCCACCCTCTTGTCTCCCCTACCCCAG
GTGTTCCACCGGTGAACTGGTAAAAATATCTCCTTCCTCAATTTCGACCTGGCAAC
CATAGGACAGTTCACCCGCTCCTAGAGGACGCGTTAGACTTGACCTGTGTTCCCC
ATCTTGGTCCCATTACGTCAGAAGCTGTGTTTCCATAAGCCACACACCAGAGATC
TCCTCCCACACAAACCAGTGGTTCTATAGAGCCTTCTTTGATACATATTCTACCTG
TGGGGCAGCCATGCTCACTGAGACGTTTGGATGATTGCCTCCTGGCTCTTCAGGG
GTACTGTGTGAGCTAAGGTTCCAGACCTAGTGCTGTGGGAATGGACAAGTTTACC
TCCCTGCTCTGTAAGCTGCATATCCTGTCACACTGAGTCAGGTCCAACCTAGCCAT
TCAGTGACTCTTAACCCTGGCTCCACGCTGACTTTATCATACACACTCGGTGACGG
CCTGGTGCAGCATTCAGCGTCCTCATGCCAGTCTGTCTGCTTTTCTAGAGCATCTC
CATGAATGAGACTGGCAGTAGCCTGCTCCACTTCTTCCTGCGCTTTGTCCGTCACA
GTGTCGTGAAGATCGATGGCAGGTAGGCCCAGACATGGGGCAAGTGGGCAGAGT
GGCGGAGGCAGGACAGGTACAGGTAGGTAGGGGCTACGCTGGCTGCTGAGCAGG
GTGCGTGCTGACTGGCCACAGCACCTAGAACTCTCGAGTGACTGCCTCCTCCTCA
TTCCTCCCTCCCTGCCTTGCCCTTCTGCTGGCTTTTGGGCCACAGAGCTGCCTACC
TATGAGACCTTAGGAGCCCCGGGATAAGCAAGAGTCCAGTGAAGTTAATGATTTT
GTGTGCCTCGAGCTCATGTTGCCATTTTGTCAGTGTCACCTCACCTGTGCAGCCAG
GCATGGGGCACATGGATCACCAAAGAAGAGAGAACAGTGCCCGGGACTGAGTCC
GCCCAAGACTACCATGAGTTACAAGCCAGACCAGGCACTCTTTTTTTTTTTATTC

TTTTCCACAAACAATTTGGCTTTATGACAGAGGTGCAGAATGGCTTGGCGTGTGT
AGTTCTGTAAACACACATACTGACACAAATGCACCTAGACGCAGTAAAGGGCTTT
CGACGATAGAACTCAGAGGCTCGTGTACTGGCTGGTTTTGTGTGTCCACTTGACA
CAAGCTGCAGTTATCACAGAAAAGGAGCCTTGGTTGTGGAAATGCCTTCATGAGA
CCCAGCTGTAAGGCATTTTCTCAACTAGTGGTCCTCAAGGTGGGGAGGACCCAGG
CCATTGTGGGTGGTGCCATCCCTGGGCTGGTAGTCTTGGATTCTATAAGAGAGCA
AGCTGAGCAAGCCAGAACAAGTAAGCCAGTGAGTAACATCCCTCCATGGCCTCT
GATCAGCTCCTGCTTCCTGAACCGCTAGAGTTCTAGTCCTGACTTCCTTTGGTGAT
GAACAGCAATGTGGAAGTGTAAGCTGAATAAACCCTTTCCTCCCCAACTTGCTTC
TTGGTCATGTTTGTGCAGGAATAGAAACCCTGACTAAGATACTCTGTAATGTAAC
ATTTACAAAATATGGAACACACTGTAAACTAATTAAGCTTATAACCCATTTTCCA
GGAGAAAAAAAGAACAAACAGGTACTCTTTTAAAAAGCACTGCTAGGGTGAAGA
GAGCAAAGTCGCTCTGGGCTGGAGCTGCCCACCAGCCCGCAAAGGCCTGTCTGGT
GTGTCTGGATCAGATGTGAGGTTGAGTGCTCGAGCAGTTGGTTACACCTTCTGCA
CCCAAAGTCCTCCCTCACCTGGAGACTGGTACAGTGTACTTGGCTCCTTTTTGCTG
AGCTGACAGAAACCCACCATGAGTGCCCATGCAGAACAGAATCCGAGGGTTATT
AGAGATGGAGTCAGGCAGGACAAGGAATATCTGATTATCTGAAGTCACAATATG
TGTTAGGATGTAATTGGAAATGTTCAATGAGCATGCTGGGACGCTACAAGCATGC
AACCGAGTGTCTTTTCACAGCCAAGGGAGCTCCAGAGTCCATCTGTAGTAAGCAC
AGATTCATGAGTCTATGTCCATGTGCACAGGGCCTCGGAGCTTCCTGACGGTTAC
TCTCGTAAATGTGAGGGGTGCCTCCCTGGGTGACCTCCTTGTCCTGACCATCTGAT
AACACCCACCCCTGCCAGCACCTGTGTGTCTGTACCTGCATGTCTGTCAGTTTGGT
TCCATTGTGTTCTCTGAGAAGGCACTAACTTTTTTAACACGGCTGTCACCAAGAAT
ATTCCTTCCGTAATAACCTGTTATGAATTGTCCCAGATAAATTTTCCATTCAGGAG
GCTGATTGCTTGTCATTCTTGTATAAAATTTAGGAACGCCGTGGAGTCAGTGGGC
AGGGCTGTGGTCCGCACCACCCCAGGTTGTAGCGGAAGAGTGGGAAAGGGAAGA
GATAAGGGTTATCTTCTTAGGTCTCAGCAGCTGACGGTGGTAGATACCACTGTTG
TATATGTGACCTGTGAGCATGCTCACTGAATGGTGTCTGTTTACCCATGCATGCAT
GGGGAGGGGTGTCAGGTACAGGTGTGAGACCCACCGTGGCTGCTCCTTCTGACCA
TAACTGTCACCAATACCACCAGGTTCTATGTGCAATGCCAGGGCATCCCCCAGGG
CTCCAGCCTGTCCACCCTGCTCTGCAGTCTGTGTTTCGGAGACATGGAGAACAAG
CTGTTTGCCGAGGTGCAGCAGGACGGGTAAGTGCTTCCTTTTACTGCTCACAGGG
GCTGGGTTGCCGAGTGGGTGTCGCTGTTGCTGTACGCTCAGGTGCCTTCGCTTTGT
GAGAAGCCATGAGGCGTACCAGAGCCATCGCTGTTAGGACAGGGAAACTGTCAT
TCCCTTCTTTTGAAGAATCACTTTGCTTTGCCTCCATGCATTCCTGTACTTATAGTG
TTATAGGGTGGCTCACTTGAAGGTGGGCGTTCTGTGAGACCTCTTGGTGTGTCTGT
CTTTTTTTTAGCTTGCTTTTACGTTTTGTCGATGACTTTCTGTTGGTGACACCTCAC
CTGGCCCATGCAAAAGCCTTTCTCAGGTAAGGACCATCATGCCTGGGTTTCTGCT
GTGTGATCAAGATAGAAGTATGTATGTACATATATCTGCATAAGATATAAACGTG
CTGAGGCAGCAAATTTAACTGGTTAGTAGTTCTGCATGCCTGGAGCTCATATAGT
CACCAAAACCTAGTGGCATCTCTACAAAGTGGCAGAGGCACCGCACCTGTGCAG
CCAGGCGTGGGACACCTGCAGCACCGAGGGAGAGAGTCTGACACCTGTGACTGG
TCTGGCCTGGTCTATCCCCCAAGGTCAACTCACAGCAGCCATCTGGCTCCCCCCGT
ACCATGAGTGTGGCTACTTGATGTGCTATCCACTGCCTGTGCACTCTATATTCTTG
TGACTGGTGAGCCCCTGCAGTAACTCTGTCCTACAGGTGTCCATGGACAACCCCA
CCTAGCAGCCCCTCCCGGTTGTCCTGATATTCATCCAGGACATCCTGACATTTCCC
TGACAAGGTCTTAGGGGCAAAACTGGCTTCTGGAAATTTCCTCAACAGGAGCAGC
GTCCTCTGTCCCAGTGGCCACTGCCTATAGGCCATTTAGTTCCTAAGTGCCACAGA
TGTGGTGGGGCCTTCATTGGGGGTGGCTTACTGACTTTGATAAGGATGACCAATT
GGGCTCTGGGACCCTGTACTACAAGTGCATGCAGCCTGCACAGAGCACTGGAAA

CACCAGTACCTCAGTGGTGACAACTTCTTAGGGCTCAGTTTCATTAACCACTTTCA
GTTTCTCATCTCTTGTCAGTAAAGAGAAGCCTACAAGCATCCCAGTGCCTGAGGG
ACCTCAATGTAAGGTCACTGCTCTGAAGTAAGGCCATAGTACCTGATTCTAGTTT
GAGCCACAGAAATTAACTTTACAAGCAGGTGTGCCCGAAACCCTGGGCTAGCGT
ACATACTGCAAACACAGCCTGGAATACACACTATAACCCACTAGTATGAATATTA
TGTAAGAAAAAAGACCGCTCCTGATGCAAAAGCCACACCTCAGAACACCAATGT
TTATTTAATAAGTCCAACTTCCCGGGAAGGTCTGTCCGCAAGGAAGCAGTCTATT
GATTAGTACTTGCTGATAAGGAGAAGCATTGCGCATCGACAAGCATCCCAAAGTA
GCTACAAGAAAAGAGGCTTTGGGGGTAGTAGGGAGGGGATGTTTTGCTACGTGA
TTGCTAAGATCTGACCCACAGAGTCATGGGAAACTTTCCACTTGTCTGAGGCCTG
GCTTATAGCCTTATTGCTCCACTGCTTTACAATGAAGACGGGGGCTAGGCTGCAG
TCTAGAGCAAGAGTGTCTGGTGTTCCATCACGGTACCCACAAGCATCTGCTCTCT
CGCGTCACAGAGCACCTCTGAGAACTTCTGTGGCATGTGCTGGGTGCCTCCAGGC
TCCCATAACCGATGTTCCTTCTCTTGTTTCAGCACCCTGGTCCATGGCGTGCCCGA
GTATGGCTGCATGATAAACTTGCAGAAGACAGTGGTGAACTTCCCTGTGGAGACC
GGCGCCCTGGGAGGTGCAGCCCCGCACCAGCTGCCTGCTCACTGCCTGTTTCCCT
GGTGTGGCTTACTGCTGGACACTCGGACTTTGGAAGTATTCTGTGACTACTCAGG
GTAAGCACACAGGGGAGTCATGAGGTCATGGGAGCAGACGGTGCTGTGGCTACG
ACTTTCTGTTTCCTGCTTTTAGACACAAGTGACCGTGAGGCCTGAAGTGATAGAG
GCCGAAAGGTGTTTCCCTCCTTTGTGGAGATGGTCACGGCCAATATAAGAACATC
TTCAGCCTTGTCCCAAGGCTCCTGCAGACCATGGAGCCAAGATGTTCCTCTATAT
GAAGTATAAGGGGCGGGGAGGGGGTGGGTGTTGGTCTCCACCTACCAAGAGCTC
ACAACCCAGATTGTAGCTCAATGTAGATATGACAGATTTGGGATAGGCACAGGTG
AAAAGAGTTGGACCATTTCTTCTTATGAGTGAAGAGTATGAAGATTCTGACATTA
CGGCAGGAGCCTGGCAACCCACCATCTTGACAGCTGTCCATTCTATGCTCAGACT
CCCCATCTCCATTGTGTTCTTTGGAGGGTCCCATTCTAAGGGAAGGGTTGGGTGC
AGATAAGCCGTCTGTACCGTGCTCTCGCAGGCCTGGGCTCCTCAGCCTCAGTCCA
TGGGACTCAGTTCAGATTTCTTGACTCCACCCTCTGCCTCCCAGACCCACCTTTGT
TGCCCCAGCCCCAGCCTAGTGCTGAGCTCGCCAGTGTACCTGCCCCAGCCCCTGG
TACTCTTCAGTGTTACAGAGTCATGGGTAAATCAATCGAAGAAGAATTCCCCTCT
GAACATACTGCTAGAGTCTGAAAACCACACACCCTTATAGGAAGCCAGGCGGGC
CCTGGACAGGGGCCATTAGCGACCATATGTAGACCATAAGGGCCCTGTTTCCAGA
GTCCATGTTAAGTGTGGCTGTGAGCTCAGCCTGAGCTTTTGGTGTGGGTGCATCA
GCCGAGCTCAGTATCCTTCTGCCGGAGCCAGGGACAATGCATCTGGAGGTCAGCC
AAAGTGGCTAGCAACATCCTAAAGGGAAAACACAGTGATGGGAGTTTCTTCTCCC
CAAACCACAGCTTTCATGCAAAGTGTGAAGAGAGCTGACAGCACCGTGGAGTGT
CACGGAGTGGGAAACCTCTTCCTCCACCCACACGCACAGGCATGCACCTCACACA
TGCACAAAAAGTAAGAAACCTGACAGAGGTAACCATACCCGTCACTTGTATTCAT
CTTTGAGGATGATGCCAAGTCACTGGACAACAAGAAGGCACCACCCCATCATTCT
GTCCTGCACGCGTCCATCCGTCGTCCCAGAGTGACCAACTCTGCAGGTGGTTTGA
CACCGCTGTAGGCACTCCCTTACTGTTTCCTGTCACCAAGATCTGGCTGGAGACA
ATCCTCCACTCCAGTCATAGCCCTGTGCTGGGAAGGTGCTCTGTCAGGCTGCAGC
CACTTAAAGAAGCACAGGGTTTGCGACTTCCTGTAAGATTCACAGTTCTTTCTGA
ATTTTCACAGCTCTCTTTGCCACCTGTGAGCAAAGATGTCTGGTAATCCCAAGCTC
AGATAAAATGGATGTTGTTCACTACATGGTTGGGCTTGGTTGTCCAGGGCTTCTA
GTGGCGCTATAAGCAAGTACCAAAAGCTGGAATGGGCCCCATGTCTGAAGGTCCT
ACCCCATCTAAGCCTGCGGCGTGTGGCAGAGACGAGAGGAGGAAGGGCAGTGGT
GCTGGTTTCTCAGGACGAGCTAGTGCAGCTATCACACAGGATACCTTTGTGCCGC
AGTTACGGACGGACCTCAATTAAGATGAGCCTCACCTTCCAGGGTGTCTCCAGGG
CCGGGAAGACCATGCGGTACAAGCTCTTGTCAGTCTTGCGGTTGAAGTGTCATGG

TCTGTTTCTAGACTTGCAGGTAAGCAGACAGGCAGCCACCTCATCGGTTGGATCT
TAGTATAGGTCCTGTTCTTCTGTGTGGGGGGACACGTTCATGGATATGCATGTGTG
TGCACAGGCGTGAGCCGTGTATGTGGGGAGCACGTAGGAAGGTGGATGTGCACT
GAAGAGACCGAAGAGCCTTGAGCACTTACCGTGCTCATCTCCGTCACTTTTCAGT
ACTGACATCCAGCTTCCCTGGATCCTGACCTTGGCTTTGGAGTCTTAGGCCTCAGC
TCTCTCCTGCTTCCTCCCCATGGGGGCCTGGCTCGATTTTCTGTTTTCCCCACACGT
GGCTTTCTTATCCTACTTGACAACACAGAAAATATTCATTTGTGATCTCGGGGACA
TTGCTGACGCTTCTCTTACCCTTGACTCCAGGTGAACAGCCTGCAGACAGTCTGCA
TCAATATATACAAGATCTTCCTGCTTCAGGCCTACAGGTGAGTCAGCGGCCGTGC
CGCCACTAGAGGGAGGCACAGGCCAGAAATGCCAGTCATGCTGTTTGCTGGGGC
TCTCAAGCTCAAGGCTGTTTGCTCCCCCAGATTGGGTGGCATGCTGAAATGCGGT
CAGGAAAAAGCAGCATTTAGTTGAGTCTCCACACAGGCCCGTATTTGTAGGGTAA
GAACCTCTTGTGGTCTGCCTGTGCTGGGAGCACATGTAACCTTGTGTGCTGACCA
GAATGGTCTTGAGACAGGAGGGGATGTTGGCATAGAAGCCCCCAAGAAAGCCCC
TCAGAAGAAATAGGATGGGAAAGGGGAGCAGGGAGATTCAGAGTCCCAGAAAG
CAGAGAACCCAGTCTTACACCAGAAGGCATGACAAGCACCTAGCTGAGAGCAGC
CTGGCCATCATGGTGCCCCACACATGGGCTTGTGCCTCTGGAACATTCTTGTCCTC
CATTGTCTGCACCTTAAGTCAGGTGCATTAGGCTAGACCCATGTCGTTCATTCACT
ACACAGTCACTTCTCTACTAACCATAGGCGCACAGCATAGGCCCTTTCTTCTGAG
GGGTGGGATTGGTTTCCCTTGACTCATGAAAGGAGAGCCGTAGCGGAGGGCACC
ATTCTGCCAGGAGTTGGCAGATTGACACACCAGCCACAGGTCAGATTTATGCCAC
TGTGGATGTGGCTGCCTCTTACATAACCTTCTGGTCACTGGGGCTGGGTGTGGGG
AAGAGCTTGTGCTGTAGAAGACACAATTTCAGTGTTGGGCAGTCTACCCCCGATG
TGATAATGTTGTAACAGGTGCAACGGGGCACCCAGGAAGGGACTAATTACATGG
CACTATACAGGATTGAAGATATGTGCGTGGGCACATGCACACACACACACACAC
ACACACACACACACACACACACACACGTTTGTGTGTTCACTTGTAGTTGTTAGGT
TCATTCGTACATGCAGTTCTATGATGTGCTGCACATAAATCTCTGTATATAATTCA
CATGTATGCACAGATAAGAGCTTGCACTTGTGTACATGCATGTGAACACTTATCA
GGTGTTCTTCCCCACCACTGTGGTTCCCGCCCAGGTTCCATGCATGTGTGATTCGG
CTTCCCTTTGGCCAGCATGTTAGGAAGAACCATGCATTCTTTCTGGGCATCATCTC
CAACCTAGCATCCTGCTGCTACGCCATCCTGAAGGTCAAGAATCCAGGTGCGTAG
ATGACCGGGGACCAGCATTCCGGTGGAGGGATGGGCATCCTGCACTCACTCGGG
AGCGAGTGTAACCAACCATCATTCCTGGGCCTCAGCCTTAGCACATTGGGGTTGT
CGAGAGCTCCTCGGAGGCCATGAGGGTCTGTGGCCAGCAGGTTTCTTCAGCCTCT
GTCTGGTCCCCTAGGAGTGTCACTAAGGGCCAAGGGTGCCCCTGGCTCCTTTCCG
CCCGAGGCCACACGTTGGCTCTGCTACCAAGCCTTCCTGCTCAAGCTGGCTGCTC
ATTCTGTCACCTACAAGTGTCTCCTGGGACCTCTTAGGACAGGTGAGTGAGCTCC
GTCCCCTCAGACAGTGAGTGCCCAGGTGAACCCAAAGGCAGACTATACATGTGCC
CATCTCCTGGGGAGGGGCCCACTCACAGCTTCCTCCTCCTCCTTCCTGCTCCCTCT
GTGCTGACTCTTATTTCAGGAGAGGCCCATGGGTGGACATGATTCACTCAGGAAC
CTCCTGATCCCTGCCTGGTCCCTTCACACCTCTGCTTGGCTGCTTCCTAGATGTGG
GGTGGGAGGAGAGGTGTTTGGGGTGGTCCTCAGGTTCCTGTCTTGTTCCTGTTTCT
GGATTGCCAATCTTGAGGTCCTTGTGTGCCCACTGTGGTGATGGTGGTGGGTTCCT
GGGTGGGACTCCTGCTTATGCCTGGCCTACATATGGCTGGGACTTGACCCAGGAA
GGCTAAGGATCTACCTCTTTGGAATGAAAGTTGAGTACATCTTCTGTTGCAGCCC
AAAAACAGCTGTGCCGGAAGCTCCCAGAGGCAACAATGACCCTCCTTAAGACTG
CAGCTGACCCAGCCCTAAGCACAGATTTTCAGACCATTTTGGACTAACCCTGTCC
CCTTCGGCTGGATGAACATGGGCCTTGTAGCCTCAGTGGTGGATCTATGTCACAA
GAGGGACTGGCCTATTGTGAAGCTAGGTCGTCCTCCAAAACCTCTGTGCCATGGG
TAGTAGTATGGGAGCTTGTCCCAGTGCCTTGTTCCCTGTAATAGGCTTGATTTCTT

TCCTGATGCCCTGGAGGAAGCAGATCCCACCCCTTTTAGTGGCAGGGATCCACTA
GCACTAAGAGCAGTGCACATAGTGCACATTGGCGCTGGGACAGTGGACAGGTGT
GATTCCTGGGCCCTGGAGTCTTCACAGCTAACCATGGAGCCTTTCACAGTACATC
CAAGCGTCTCAGAGATGAAACAGGACAGCAACCTATTGCAGCCTGAACATACTCT
GAACTCAGGCGACTGCCTTAGCTACTTTTATACTGCTGCAATAAAACAGCAAGCC
AACTTACAAAAGCAGGATCTTTCTACTGGAGCAGTATCTCTGAGAGTTTACGTCT
TGATCCGTAAGCACGAGGCAGAGAGAGAGAGAGAGAGAGAGAGCTATCTGGGA
ATGGTGGGAGTCTCCTTTTTGTTTCTTTTTAAACCTTAATTTTTCAGTGGCAGTCTG
ACAGACAGTTAGGAAAGGTGGAGGTTGAAATCCAACAGGTCACAGTGAAATCCA
AAGGGATCACACTAGGGATATTCCGTGACTTCTCGGAAGCTAAGTTTCCTCGGCA
GCATTTGACAGTAACCATGGTGGGTGTCTACCAAGATCACTGTGAGGATAAAATA
GGGTAAAGTGTATTTGTACTGAAGTGGAAAGTCACATGCAAATAAAGAGTAAAT
TGTGGAA
