# Supplementary material for: Tert Deletion Impairs Circadian Regulation of Blood Pressure in Male Spontaneously Hypertensive Rats
Source: Hypertension. 2025 Dec 10;83(2):e25510. doi: 10.1161/HYPERTENSIONAHA.125.25510 (PMC12822765; doi:10.1161/HYPERTENSIONAHA.125.25510)
Supplement: Supplementary file 2 [file hyp-83-e25510-s002.pdf]

*** ARRIVE Reporting Guidelines**

This study involves use of animal models:

Yes

Manuscripts reporting on animal research are expected to adhere to the [ARRIVE] guidelines. A completed ARRIVE reporting guideline checklist will be required prior to acceptance and the information should be clearly presented in the manuscript. The following shortened version of the checklist should be completed at this time; a completed ARRIVE checklist is required to be uploaded during revision submission.

Animals

Species, age, sex, strain, and sources of animals are described:

Yes

Randomization

Randomization and allocation concealment were performed and described:

N/A

Blinding

Blinding was performed and the process described:

N/A

Inclusions and Exclusions (a)

Specific criteria for inclusions and exclusions are specified:

N/A

Inclusions and Exclusions (b)

Criteria for inclusions and exclusion were set before the study:

N/A

Reporting of Excluded Animals

All animals excluded after the randomization are reported:

N/A

Statistical Methods

Statistical Methods are described:

Yes

Date completed: 11/18/2025 03:25:27

User pid: 1013819
